# Hypoxia-inducible transcription factors: architects of tumorigenesis and targets for anticancer drug discovery

**DOI:** 10.1080/21541264.2024.2417475

**Published:** 2024-10-29

**Authors:** Alexander McDermott, Ali Tavassoli

**Affiliations:** School of Chemistry, University of Southampton, Southampton, UK

**Keywords:** Hypoxia-inducible factors, hypoxia, HIFs, HIF-1, HIF-2, cancer

## Abstract

Hypoxia-inducible factors (HIFs) play a pivotal role as master regulators of tumor survival and growth, controlling a wide array of cellular processes in response to hypoxic stress. Clinical data correlates upregulated HIF-1 and HIF-2 levels with an aggressive tumor phenotype and poor patient outcome. Despite extensive validation as a target in cancer, pharmaceutical targeting of HIFs, particularly the interaction between α and βsubunits that forms the active transcription factor, has proved challenging. Nonetheless, many indirect inhibitors of HIFs have been identified, targeting diverse parts of this pathway. Significant strides have also been made in the development of direct inhibitors of HIF-2, exemplified by the FDA approval of Belzutifan for the treatment of metastatic clear cell renal carcinoma. While efforts to target HIF-1 using various therapeutic modalities have shown promise, no clinical candidates have yet emerged. This review aims to provide insights into the intricate and extensive role played by HIFs in cancer, and the ongoing efforts to develop therapeutic agents against this target.

## Introduction

A low oxygen microenvironment (hypoxia), results in an intricate interplay between cellular adaptation and pathological progression [1]. The initial cellular adaptive responses primarily involve transition from oxidative to nonoxidative metabolic pathways, e.g., the promotion of glycolytic metabolism in ischemic tissues [2].The drivers of these cellular responses are hypoxia-inducible factors (HIFs) which are a family of transcription factors that regulate hundreds of genes to facilitate cellular adaptation and survival under oxygen-deficient conditions [3,4]. Genes regulated by HIFs participate in an array of biological processes including glycolysis, angiogenesis, erythropoiesis, apoptosis evasion and immune modulation [5–9]. Consequently, prolonged HIF activation has been associated with the development of numerous diseases, notably cancer [10,11]. In recognition of their ground-breaking work uncovering the molecular mechanism by which cells utilizes HIFs to sense and adapt to changes in oxygen levels, William Kaelin Jr, Sir Peter J. Ratcliffe and Gregg L. Semenza were awarded the Nobel Prize in Physiology and Medicine in 2019 [12].

The accelerated growth of tumors induces abnormal vascular structures including nonfunctional blood vessels results in a diminished oxygen supply to tumor cells giving rise to a hypoxic microenvironment (HME), to which cancer cells adapt via HIF activation [13–16]. HIF activation plays a pivotal role in cancer cell adaptation and progression, encompassing key cancer hall marks such as rapid cell proliferation and metastasis [17,18]. Tumor hypoxia is thus associated with aggressive tumor phenotypes, treatment resistance (chemo and radiotherapy) and poor clinical prognosis [19–22]. Clinical evidence unequivocally shows the need for therapeutic agents that target HIFs, aiming to disrupt the adaptive responses of tumors within HMEs with the goal of improving patient prognosis [11].

Over the last three decades, extensive efforts have been made to unravel the multifaceted biology of HIFs, shedding light on their involvement in cancer development, and highlighting the need for inhibiting their function in tumors [23]. In this review, we delineate the functional intricacies and regulatory mechanisms governing HIF isoforms and highlight their role as master regulators in cancer development. We discuss the existing strategies for inhibiting HIF, particularly those involving inhibitors that directly interact with these transcription factors, and the challenges associated with this approach.

## Hypoxia inducible factors: structure, regulation, and isoform function

HIFs constitute a family of transcription factors that modulate the homeostatic cellular response to hypoxic conditions. To date, three HIF transcription factors have been identified: HIF-1, HIF-2 and HIF-3 with HIF-1 and HIF-2 being the most extensively investigated. The discovery of HIFs dates to 1991, when Semenza and colleagues identified a hypoxia inducible enhancer region located 3’ to the human erythropoietin (*EPO*) gene [24]. Following subsequent investigations, Semenza et al. identified HIF-1 as the nuclear factor responsible for binding to this enhancer element, thereby facilitating the upregulation of *EPO* in hypoxia [25]. Shortly after, Ratcliffe et al. revealed the ubiquitous nature of oxygen-sensing in multiple mammalian cell lines and provided the first report of glycolytic enzymes being regulated by HIF [26,27]. The HIF-1 protein was later isolated by Semenza et al., who verified that HIF-1 exists as a heterodimer consisting of HIF-1α and HIF-1β [28]. Prior to this discovery, HIF-1β had already been identified as a binder to the aryl hydrocarbon receptor and was classed as the aryl hydrocarbon nuclear translocator (ARNT) [29]. The HIF-2α (also known as EPAS) and HIF-3α isoforms were subsequently identified through DNA homology searches on HIF-1α and screens aimed at identifying partners of HIF-1β [30–33].

**Figure 1.**
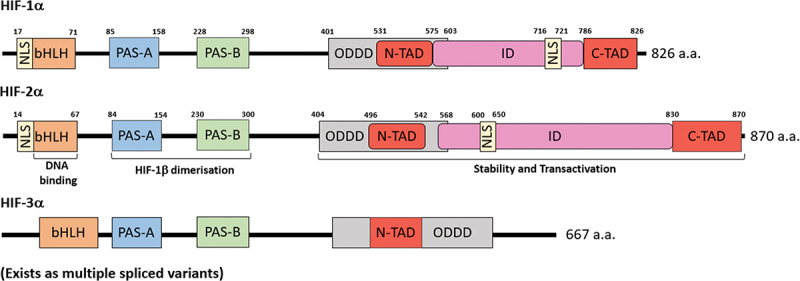
The domain structure of HIF-1α, HIF-2α and HIF-3α. The α-subunits contain a basic HLH domain responsible for binding DNA followed by PAS-A and PAS-B, of which facilitates dimerisation with HIF-1β to form a stable heterodimer. The ODD domain affects the stability of the α-subunit, whilst the ID domain effects the transcriptional activity of the protein. Situated in this region are TAD domains of which N-TAD imparts gene specificity whilst the C-TAD recruits CBP/p300 transcriptional co-activators. It should be noted HIF-3α lacks the C-TAD and exists as multiple splice variants. There are also NLSs, responsible for importing HIF-α subunits into the nucleus in hypoxic conditions.

The α and β subunits were found to be within the basic helix-loop-helix (bHLH)-PER-ARNT-SIM (bHLH-PAS) protein family of structural domain transcription factors, which contain a bHLH domain and two PAS motifs (PAS-A and PAS-B) [34–36]. These motifs play a crucial role in mediating the heterodimerisation between the two subunits [37]. Concurrently, the basic region following the N-terminal of the bHLH domain is responsible for binding to the hypoxia response elements, triggering the expression of genes regulated by hypoxia [38–41]. HIF-α subunits possess unique structural elements, including the oxygen dependent degradation domain (ODDD), located at the C-terminal (Figure 1) [42]. This is a proline-serine-threonine rich protein domain that mediates HIF-1α degradation in an oxygen-dependent manner. Overlapping the ODDD is the NH_2_-terminal transactivation domain (N-TAD), one of two terminal transactivation domains (TADS) that function synergistically to facilitate optimal transcriptional activity [43–45]. The N-TAD imparts specificity in gene binding, whist the COOH-terminal transactivation domain (C-TAD) plays a pivotal role in recruiting transcriptional co-activators, including CREB binding protein (CBP) and p300, both of which are histone acetyltransferases (HATs). In addition to these co-activators, other co-factors such as the mediator complex and TIP60-containing chromatin regulatory complexes, also play a critical role in modulating gene expression [46,47]. Interestingly, despite the structural and functional similarity of the C-TAD in HIF-1α and HIF-2α, the HIF-3α isoform lacks the C-TAD domain, suggesting a divergence in its mechanism of transcriptional regulation [48]. Situated between the TADs of HIF-1αand HIF-2α is an inhibitory domain discovered to deactivate the transcriptional activity inherent to these domains. These subunits also contain two nuclear localization signals (NLSs) on the N and C terminal, which are responsible for importing HIF-α subunits into the nucleus to initiate transcription in hypoxia (Figure 1) [49].

**Figure 2.**
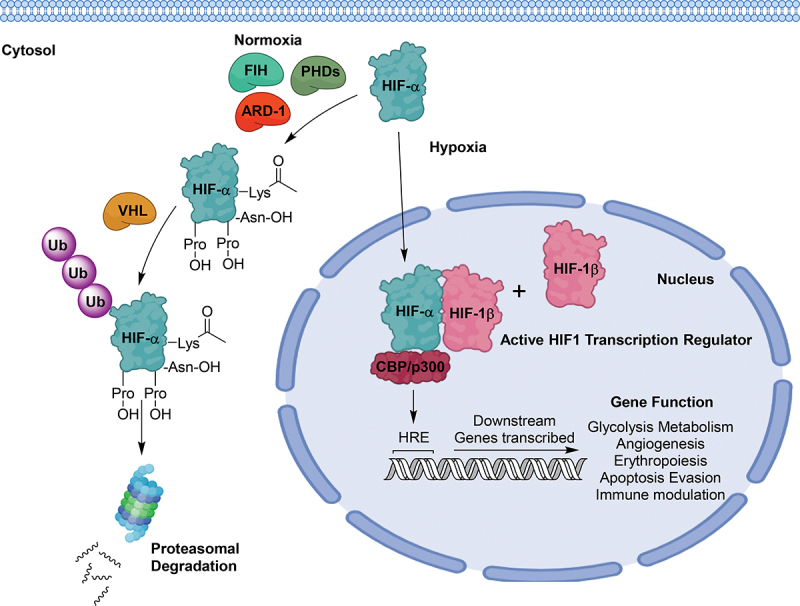
The oxygen dependent regulatory pathway of HIF-1α subunits. During hypoxia, HIF-α subunits are imported into the nucleus where they dimerise with HIF-1β and recruit transcriptional co-activators. This active transcription complex then transcribes genes that enable cells to adapt to HMEs. During normoxia, HIF-α stability is controlled by several oxygen-dependent proteins. PHDs hydroxylate HIF-α subunits on specific proline residues, which are recognised by the VHL E3 ligase. The VHL poly-ubiquitinates the HIF-α subunits, targeting it for proteasomal degradation. ARD-1 aids in this process by acetylating a specific lysine on the α-subunits, stabilising their interaction with the VHL E3 ligase. FIH-1 is involved in a separate inactivation pathway whereby it hydroxylates a specific asparagine residue on the C-TAD domain, preventing the interaction of CBP and p300 co-factors.

Whilst the HIF-1β is constitutively expressed, the regulation of the alpha subunits is tightly controlled to ensure an efficient response to hypoxic conditions. Under normoxic conditions, HIF-1α/2α are negatively regulated by the ubiquitin proteasome system (UPS) initiated by Fe (II)-dependent prolyl hydroxylases (PHDs) (Figure 2) [50–53]. Two proline residues within the ODDD are hydroxylated by the PHDs (P402/P564 in HIF-1α and P405/P531 in HIF-2α) in an oxidative decarboxylation reaction involving oxygen, 2-oxoglutarate, ascorbate and iron as a co-factor [50]. In mammalian cells, three isoforms of PHDs, namely PHD1, PHD2 and PHD3, have been recognized to co-ordinate this protein modification, with PHD2 found to play a dominant role [53]. Using short interfering RNA (siRNA), Berra et al. demonstrated that “silencing” PHD1 and PHD3 did not impact HIF-1α stability whilst “silencing” of PHD2 alone was sufficient to stabilize and activate HIF-1α in normoxia. Hydroxylation of the proline residues is imperative for recruiting the von Hippel Lindau (VHL) protein, where the 4-hydroxyproline establishes a highly specific interaction with a surface pocket on VHL [54,55]. Subsequently, VHL recruits additional proteins, including elongin C, elongin B, cullin-2 and Rbx 1 to form the VCB-Cul2 E3 ligase complex [56–58]. The recruitment of this E3 ligase complex induces ubiquitination of the alpha subunit, ultimately leading to its degradation via the 26s proteasome (Figure 2) [59,60]. Notably, HIF-1α contains a lysine residue in the ODDD, which is acetylated by the acetyl transferase protein-arrest defective 1 (ARD-1) (Figure 2). This acetylation stabilizes the interaction between pVHL and HIF-1α, facilitating the ubiquitination of HIF-1α [61]. Independent to UPS degradation, the transcriptional activity of HIF-1α and 2α are regulated by the hydroxylation of an asparagine residue (Asn-803 in HIF-1α and Asn-851 in HIF-2α) located on the C-TAD [62]. This modification is catalyzed by an asparagine hydroxylase, Factor Inhibiting HIF-1 (FIH-1) which prevents the C-TAD from interacting with CBP, p300 and other co-factors, inactivating the alpha subunits [63,64]. HIF-1α exhibits higher hydroxylation sensitivity to FIH-1 than HIF-2α. Bracken et al. found that a conserved Ala-804 positioned proximal to the hydroxylation site of HIF-1α forms an intramolecular hydrogen bond to a conserved Val-802 [65,66]. This interaction significantly impacted the catalytic activity of FIH-1 whilst not affecting the binding between HIF-1α and FIH-1.

Beyond oxygen-dependent degradation pathways, regulatory mechanisms independent of oxygen play a significant role in the stabilization of HIF-α subunits. A notable example is the pathway involving the receptor of activated protein C-kinase 1 (RACK1). In this process, RACK1 competes with Heat Shock Protein 90 (HSP90) for binding to the PAS-A domain of HIF-1α, subsequently recruiting Elongin C, Elongin B, and additional subunits [67]. This recruitment results in the ubiquitination and degradation of HIF-1α in a PHD/VHL-independent manner, establishing an oxygen-independent proteasomal degradation mechanism. Other oxygen-independent degradation pathways involve the interaction between HIF-1α and p53, mediated by the MDM2 E3 ligase, which inhibits transactivation and promotes degradation [68,69]. The Hypoxia-Associated Factor (HAF), a multifunctional DNA binding ubiquitin ligase, also induces ubiquitination and degradation of HIF-1α irrespective of oxygen tension [70]. The Carboxyl Terminus of Hsc70-Interacting Protein (CHIP), an atypical E3 ligase, also degrades HIF-1α independently of hypoxia/normoxia, with the interaction stabilized by the presence of Heat Shock Protein 70 (HSP70) [71,72].

The stringent regulation of HIF-α isoforms ensure an effective cellular response to hypoxia. Upon heterodimerisation with HIF-1β (ARNT) and co-factor recruitment, both HIF-1α and HIF-2α activate HRE-dependent gene transcription for cellular adaptation to hypoxia conditions. HIF-1α and HIF-2α share highly conserved domains, engage in similar protein–protein interactions (PPI) for activation, and bind to the same HRE binding site, they display differences in gene expression patterns, tissue distributions, and implications in cancer [35]. HIF-3α is thought to act as a regulator of the other 2 HIF-α isoforms, although its role remains enigmatic, primarily due to its existence in multiple spliced variants [73].

HIF-1α is ubiquitously expressed in mammalian cells, whilst there is evidence to suggest HIF-2α exhibits a more tissue selective distribution. mRNA hybridization assays revealed preferential HIF-2 expression in the endothelium and highly vascularized tissues, consistent with observed elevated HIF-2α levels in regions with high Vascular Endothelial Growth Factor (VEGF) expression [31,33]. Subsequent investigations in human tumors demonstrated a positive correlation between the expression of HIF-2α, tumor vascularization, and growth [74,75]. Further studies identified widespread HIF-2α distribution in specific cell lines across mammalian organs. Although both HIF-α isoforms are expressed in these organs, there is variability in cellular expression overlap. Up-regulation of HIF-1α and HIF-2α has been reported in hepatocytes, cardiomyocytes, and myocardial endothelial cells, while the kidney and brain exhibited isoform induction in different cell populations. HIF-2α was confined to non-parenchymal cells in the kidney and brain, whereas HIF-1α was expressed in renal tubuli and neuronal cells [76]. Such data underscores the complementary roles of HIF-α subunits, emphasizing their non-redundant and potentially overlapping functions. This observation aligns with early evidence from gene ablation studies in embryos, where knockout of both HIF-αisoforms resulted in different embryonic malformations and lethality [77–79].

Investigations have revealed both shared and unique gene targets of HIF-1 and 2 isoforms *in vivo* and across multiple cell lines, further characterizing their functional roles. Genes induced by hypoxia and encoded by HIF isoforms encompass those involved in glucose transportation (GLUT1), lipid metabolism (ADRP), pH homeostasis (CAXII), angiogenesis (VEGF), cytoskeletal structure (filaggrin) and immune cytokine (IL-6) [80,81]. Genes exclusively regulated by HIF-1α are predominantly involved in regulating glycolysis (PGK & LDHA), pH (CAXIV) and cell death (BNIP3). In contrast, HIF-2α uniquely controls cell cycle progression (cyclic D1), redox metabolic sensors (OCT4) and growth factors (TGF-a), extending HIF-2α’s function beyond vascular regulation [81–86]. The molecular mechanism delineating isoform specificity in hypoxic gene regulation remains an on-going area of study. A notable study that sheds light on this mechanism by Hu et al. investigated how each domain of HIF-1α and HIF-2α contribute to specific gene regulation. Employing a strategy involving multiple deletions and domain swap mutants of these proteins, they highlighted how the N-TAD domain is crucial in governing gene selectivity among these isoforms. Additionally, their findings emphasize the importance of the co-regulator ELK in the activation of HIF target genes, suggesting that differential interactions with transcriptional co-factors are probable determinants of varied gene activation, a principle corroborated by several groups [44,87–89].

## Hypoxia inducible factors in carcinogenesis

Human cancer biopsies obtained from most primary human cancers and their respective metastases exhibit elevated levels of HIF-1α or HIF-2α, or both proteins [90]. This observation has prompted intensified research efforts aimed at unraveling the intricate molecular mechanisms underpinning HIF-driven cancer and understanding the roles of the specific genes they regulate. These investigations have unveiled the pivotal roles of HIFs in various aspects of cancer biology, including angiogenesis, metabolic reprogramming, cell proliferation, invasion, metathesis, epithelial–mesenchymal transition, immune evasion, and therapy resistance. In the following section, we discuss the circumstances that lead to HIF activation in tumors and explore the intricate roles and regulating mechanisms of the two major isoforms in cancer progression.

### Mechanisms for HIF activation in tumours

Intratumoral hypoxia is pervasive in solid tumors, stemming from multiple causes, including: 1. Perfusion related (acute) hypoxia arises due to abnormal structural and functional tumor vasculature induced by the rapid proliferation of tumors, resulting in ischemic hypoxia [91]. 2. Diffusion-related (chronic) hypoxia occurs when tumor cells are over 70 μm away from nutritive blood vessels, hindering sufficient oxygen delivery via diffusion [92,93]. 3. Anaemic hypoxia results from reduced blood capacity to effectively transport oxygen, often correlated with low hemoglobin levels (<10-12/dl) or by therapy-induced anemia [94]. In these conditions, the principal regulators of HIF-α subunits including PHDs and FIH are inactivated, resulting in their stabilization. Consequently, HIF-1 and HIF-2 transcription factors become active, facilitating adaptation to the harsh tumor microenvironment. Intratumoural hypoxia has been linked to induced HIF-1α overexpression and aggressive, cancer phenotypes. Extensive immunohistochemistry analysis of tumor specimens from various cancer types shows prevalent HIF-1α overexpression in most advanced cases, and unfavorable prognosis for patients [95–99].

HIF-1α and HIF-2α can be upregulated in normoxic conditions due to the loss of function of various tumor suppressors, independently of intratumoral hypoxia. A noteworthy example is observed in individuals with VHL syndrome, a hereditary disease characterized by a heterozygous germline loss-of-function mutation in the VHL tumor suppressor gene [100]. The inactivation of the second allele of the VHL gene in tumor tissue is correlated with benign and malignant tumors, affecting various systems, including the central nervous system and visceral organs [101,102]. Prevalent tumors linked to VHL syndrome encompass hemangioblastomas, pheochromocytomas, paragangliomas and pancreatic neuroendocrine tumors and clear cell renal cell carcinoma (RCC). RCCs are often characterized as the most common and aggressive form of cancer associated with VHL syndrome.

Other tumor suppressors, such as p53, regulate HIF-1 activity through MDM2-mediated UPS degradation of the HIF-1α subunits. Homozygous deletion and point mutations on the p53 gene have been shown to increase HIF-1α induced VEGF expression in tumor xenografts [103,104]. Conversely, studies indicate that MDM2 overexpression increases HIF-1α levels, implying a nuanced regulatory relationship between MDM2 and HIF-1α influenced by cell type and hypoxia severity [105,106]. PTEN, another tumor suppressor associated with HIF-1 activity, exhibits germline mutations that increase the risk of developing cancer, particularly glioblastomas, highly vascularized tumors associated with high VEGF levels [107]. PTEN inactivation has been demonstrated to lead to hypoxia-inducible gene expression in glioblastoma lines through the activation of the PI(3)K/Akt survival pathway [107]. Akt, a regulator of the mammalian target of rapamycin (mTOR), was initially thought to act as a HIF-1α stabilizer [108,109]. However, other studies have contradicted this finding, suggesting that mTOR drives HIF-1α accumulation through multiple pathways, including activation of Signal Transducer and Activation of Transcription 3 (STAT3), 4E-binding protein 1 (4E-BP1)/eIF4E translation initiator factor and ribosomal protein S6 kinase-1 (S6K1) [110].

Dysregulation of receptor tyrosine kinase (RTK) mediated signaling is pervasive in human cancers and is often correlated with poor prognosis [111]. The complex crosstalk between hypoxia-related signaling and RTKs contributes to the development of aggressive cancer types associated with RTK dysregulation. Notably, the epidermal growth factor receptor (EGFR) is a prime example, where a strong correlation exists between EGFR-driven cancers and increased HIF-1α levels across various cancer types, even under normoxic conditions [112]. Among these, Human epidermal growth factor receptor 2 (HER2) stands out as a prominent example. With involvement in a third of all breast cancer diagnoses, enhanced HER2 activity or expression is linked to high tumor grades and resistance to treatment, ultimately resulting in high mortality rates [113]. Overexpression of HER2 has been found to impact VEGF expression in normoxia [114,115]. Multiple studies suggest that HER2 regulates this expression by effecting HIF-1α via the PI3K/Akt/mTOR signaling pathway [116–119]. HER2 has also emerged as a key regulator of HIF-2α in both normoxic and hypoxic breast cancer pathologies. In MCF7-HER2 cells under these conditions Jarman et al. demonstrated increased HIF-2α mRNA levels, while no expression of HIF-1α was detected in normoxia. This suggested HER2’s involvement in the transcriptional regulation of HIF-2α rather than HIF-1α [120]. The authors also conducted a thorough meta-analysis focussed on HER2 positive breast tumors, revealing that elevated HIF-2α expression is associated with poor disease-specific survival of patients.

### The HIF-1 and HIF-2 duality: the HIF switch in hypoxia response in tumours

**Figure 3.**
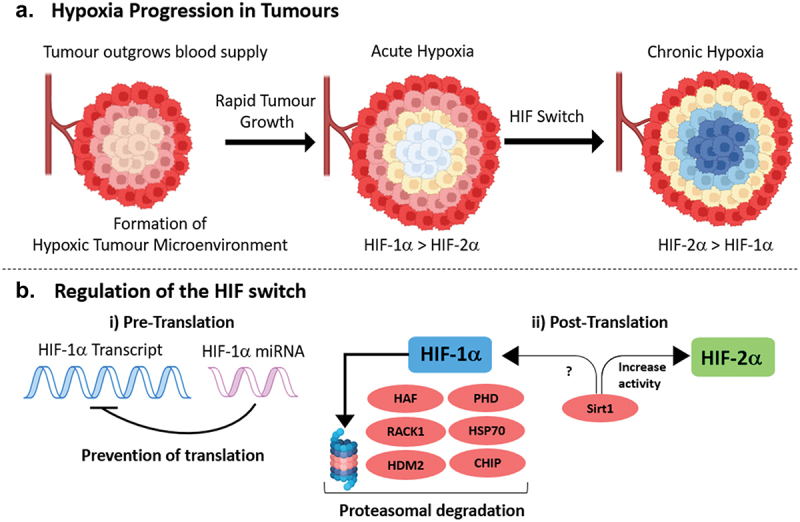
(a) Hypoxia in tumours. HMEs develop in tumours that have an insufficient oxygen supply from blood vessels. In response to acute hypoxia, HIF-1α plays a predominant role over HIF-2α in the initial adaptation of cancer cells to the HME. During chronic hypoxia, cancer cells undergo a process known as the HIF switch, transitioning to a HIF-2α mediated hypoxia response. (b) the regulation of the HIF switch. During chronic hypoxia, cancer cells transition to HIF-2α-mediated responses through a HIF switch which is regulated at pre- and post-translational levels. i) pre-translation, HIF-1α transcripts are targeted by multiple miRNAs in different cell lines preventing translation. ii) post-translation, HIF-1α stability during the HIF switch is controlled by multiple proteins that induce its degradation via the proteasome. Sirt1 has been reported to stabilise HIF-2α during this transition, whilst its role in regulating HIF-1α remains unclear.

The rapid proliferation of cancer cells into solid tumors leads to the formation of HMEs, which in turn trigger the activation of HIFs within cancer cells (Figure 3). These HIFs then upregulate numerous homeostatic genes essential for tumor survival and progression. Consequently, the collective upregulation of these genes fosters the development of aggressive tumors, ultimately resulting in poor patient prognosis. Both HIF-1 and HIF-2 play critical roles in responding to hypoxia, regulating a combination of unique and overlapping genes. The activation of different HIF transcription factors is mainly contingent on the duration of hypoxia experienced by the cancer cell. HIF-1 demonstrates rapid responsiveness to acute hypoxia, regulating the transition over to glycolytic respiration, regulating pH and initiating neovascularisation. With prolonged hypoxia exposure, cancer cells frequently transition to HIF-2 mediated regulation, promoting the maturation of a new vascular networks accompanied with elevated erythropoietin expression, thereby enhancing circulation to the tumor. Additionally, HIF-2 facilitates cancer progression by upregulating matrix metalloproteases, essential for tumor migration and invasion [121,122].

Cancer cells primarily transition from HIF-1 to HIF-2 through protein-based mechanisms, notably employing E3 ligases to selectively degrade HIF-1 via the proteasome. For instance, HAF, is downregulated in cancer cells during acute hypoxic conditions, but undergoes upregulation upon exposure to prolonged hypoxia. HAF selectively degrades HIF-1α while preserving HIF-2α levels and enhancing its transcriptional activity by binding to a region on its carboxyl terminus. Koh et al. successfully demonstrated this transition, leading to the development of an aggressive cancer stem cell phenotype *in vivo* [123]. Additionally, HSP70 has been identified to facilitate HIF-1α ubiquitination by recruiting the CHIP E3 ligase to degrade HIF-1α without affecting HIF-2α stability [72]. Similarly, both RACK1 and HDM2 contribute to the HIF-switch: RACK1 competes with HSP90 for binding, prompting HIF-1α degradation, while human double minute 2 (HDM2) selectively induces proteasomal degradation via a p53-HIF-1α interaction, with HIF-2α remaining unaffected [67,67,124]. Finally, the Stress-responsive genetic regulator sirtuin 1 (Sirt1) deacetylase has been identified to augment the HIF-1/HIF-2 activity pathway, by deacetylating unique lysine residues on HIF-2, increasing its activity, though its effect on HIF-1 remains enigmatic [125].

PHD reactivation has been identified to regulate the shift from HIF-1 to HIF-2 in hypoxic cancer cells by promoting glycolytic metabolism, which reduces the mitochondrial oxygen demand. It has been reported that nitric oxide exposure and inhibition of the electron transport chain in hypoxic cells redistributes oxygen into the cytosol, reactivating PHD-dependent degradation of HIF-1α [126]. Jaskiewicz et al. reported that reactivation of the HIF-1/HIF-2 switch in endothelial cells typically initiates 8 hours after hypoxia onset. Additionally, they observed greater stability of HIF-2α mRNA compared to HIF-1α mRNA during prolonged hypoxia exposure across multiple endothelial cell lines, contributing to the HIF-1/HIF-2 switch [127]. This stability is attributed to its 3’untranslated region (3’UTR) of HIF-2α mRNA, which is less susceptible to destabilization by AU-rich elements [128,129]. Moreover, HIF-2α mRNA lacks miRNA sequences targeting it for degradation, unlike HIF-1α mRNA targeted by tissue-specific miRNAs in various cancers [130].

The HIF switch is commonly observed in most solid tumor cases, characterized by nuclear accumulation of both HIF-1α and HIF-2α in various carcinomas, including those affecting the bladder, brain, breast, colon, ovarian, pancreatic, prostate, and renal tissues [131]. The transition is notably pronounced in individuals with VHL syndrome, who develop RCC later in life, often associated with a poor prognosis. The loss of VHL results in the inability to degrade HIF-1α and HIF-2α, causing their accumulation in cells. Several groups found that RCC is primarily influenced by HIF-2 [132–134]. They observed that the transcriptional selectivity favoring HIF-2α over HIF-1α correlated with their respective impacts on tumor growth, with HIF-2α promoting tumor proliferation and HIF-1α exerting an inhibitory effect [134]. These responses have been linked to the distinct effects of HIF-α isoforms on c-MYC activity: HIF-2α collaborates with and enhances c-MYC transcriptional activity, stimulating cell cyclic progression and proliferation. In contrast, HIF-1α binds with SP1 via the PAS-B domain, displacing c-MYC from binding multiple gene targets and hindering its role in cancer progression [135–137]. It is evident that HIF-α proteins not only control genes to influence tumor progression but also modulate other oncoproteins to either promote or inhibit cancer progression, as extensively reviewed by Keith et al. [138].

There is significant role of HIFs in cancer progression, yet the intricacies of the HIF-switch remain complex as it is influenced by a multitude of tumor-related factors. These factors encompass the tumor’s location within the body, the specific cell type involved, the unique mutational landscape of the tumor’s cells, the type of tumor microenvironment and the individual’s genetic, epigenetic, and environmental contexts [121,138,139]. This intricate interplay leads to variations in metabolic processes and oxygen availability, thereby impacting the utilization of HIF isoforms across different cancer types. Further research into the role of HIF in various cancer contexts is crucial for pinpointing optimal treatment timing and targets, leading to more effective therapies.

### HIFs as tumour suppressors

The roles of HIF-1α and HIF-2α in promoting tumorigenesis are well established and often considered hallmarks of cancers associated with poor prognosis. However, emerging evidence suggests that these proteins can also exhibit tumor-suppressive functions under specific conditions. Notably in VHL-deficient RCC, there is a pronounced preference for HIF-2α expression, which correlates with enhanced tumor growth, while HIF-1α appears to inhibit tumor proliferation. This inhibitory role of HIF-1α has been demonstrated in both *in vitro* studies and nude mouse xenograft models [134,140]. HIF-1α antagonizes the activity of the oncoprotein c-MYC by preventing its interaction with its partner protein, MAX. This disruption reduces expression of cyclic D2 while increasing levels of the cyclic-dependent kinase inhibitor *p21*^cip1^, thereby leading to G1-phase cell cycle arrest. In contrast, HIF-2α has been shown to promote c-MYC-mediated transcription, driving cell cycle progression and tumor proliferation [135,141]. However, several studies contradict the above. Analysis of human ccRCC specimens show that HIF-1α is highly expressed in renal tumors, either alone, or with HIF-2α; for example, Immunostaining of over 100 primary surgical ccRCC specimen showed the expression of HIF-1α in 84% of the samples [142]. Data from The Cancer Genomics Atlas shows that HIF-1α and HIF-2α regulate distinct networks in RCC, both of which are associated with worse overall survival [143,144]. In a syngeneic RCC mouse mode, HIF-1α was shown to promote invasion and migration [145]. The study with the largest sample size investigating immunohistochemical (IHC) expression of HIF-1α and its relationship with survival in ccRCC (*n* = 308) showed that ccRCC had much higher HIF-1α IHC expression than normal kidney tissue and that this associated with significantly worse survival, arguing against HIF-1α being a tumor suppressor gene in ccRCC [146].

In addition to its effects on cell cycle regulation, HIF-1α has also been implicated in the activation of pro-apoptotic pathways. It upregulates the expression of genes such as Bcl-2/adenovirus E1B 19 KDa-interacting protein 3 (BNIP3) and Nip3-like protein X (NIX), promoting apoptosis in hypoxic human tumors [147,148]. HIF-2α has also demonstrated to exert pro-apoptotic effects in specific contexts, such as in rat glioma models, where despite increasing angiogenesis, HIF-2α enhances tumor cell apoptosis and reduces tumor growth [149].

Recent multi-omics studies have shed light on how HIF-1α may exert tumor suppressive effects, particularly by examining the early transcriptional response to acute hypoxia and identifying specific target genes regulated by HIF-1α. It was found that HIF-1α mediated transactivation can repress MYC gene targets through the activation of MXI1, a known MYC antagonist. Furthermore, HIF-1α exerts tumor-suppressive effects under normoxic conditions, notably through the inhibition of mTOR signaling via the upregulation of DDIT4. Further analysis of human cancers identified a subset of HIF-1α target genes involved in extracellular matrix remodeling, a key hallmark of aggressive tumor behavior [150]. These findings emphasize the need for continued investigation into the gene expression patterns that govern the tumor-supportive or suppressive functions of HIF-1α and HIF-2α, emphasizing the need for nuanced approaches to HIF-targeted therapies in cancer.

## Inhibitors of hypoxia inducible factors

The recognition of HIFs crucial involvement in cancer pathogenesis has propelled intensive efforts to exploit these factors as therapeutics targets. Nevertheless, the complexity of HIF regulatory pathways, intertwined with other signaling cascades, presents challenges in designing rational inhibitors. Directly targeting HIF proteins has been especially challenging due to their reliance on PPIs to form functional transcription factors. Historically, designing inhibitors against proteins that operate through PPIs has been exceptionally challenging. PPI interfaces typically lack distinct features, with a surface area ranging between 1500 and 3000 A^2^, relative to catalytic binding pockets which contain well-defined hydrophobic cavities ranging between 300 and 1000 A^2^ [2,151]. However, across their large interfaces, PPI sites harbor several small hydrophobic cavities known as “hot spots”, collectively contributing the majority of the binding energy required for their interactions [152]. Designing drug-like molecules capable of interacting with multiple hot-spots and achieve sufficient affinity to outcompete with the target proteins partner presents a significant challenge. Thus, most identified HIF inhibitors target the regulatory mechanisms that govern the transcriptional activation of HIF-1/2 without directly engaging with α or β subunits. Many of these molecules were initially designed to target specific proteins and other cellular pathways for anti-cancer effects, with the discovery of their inhibition of HIF-1/2 function being serendipitous. These inhibitors include those that inhibit the RTK/PI3K/Akt/mTOR pathway that effect translation initiation of HIF-α subunits further upstream. The inhibitor list of this pathway is extensive with other excellent reviews covering this topic in detail. Thus far, the regulation of HIF-1/2 transcriptional activation has involved directly targeting various aspects, including HIF-α expression/protein synthesis, transcriptional activity, nuclear translocation, co-activators, stability, and HRE-DNA binding sites. It is noteworthy that due to the ubiquitous nature of HIF-1β, most efforts have concentrated on targeting the α-subunit, given its context-dependent expression.

### Inhibition of hif-α mRNA expression and protein synthesis

Directly targeting specific mRNA sequences has emerged as a successful strategy in the field of anticancer therapeutics, utilizing antisense oligonucleotides to achieve high-affinity binding to target mRNA and inhibit translation. This approach has been applied to target HIF-1α, leading to the development of EZN-2968, a 16-met antisense oligonucleotide designed to specifically bind HIF-1α mRNA. Preclinical investigations demonstrated the efficacy of EZN-2968 in selectively reducing HIF-1α mRNA levels, consequently downregulating the expression of HIF-1 regulated genes. Moreover, in xenograft models, EZN-2968 exhibited tumor growth suppression accompanied by a favorable safety profile in animal studies, prompting its advancement in clinical trials [153]. In a set of phase-1 clinical trials with patients with advanced tumors, EZN-2968 was able to decrease HIF-1α levels from biopsies though their sample size of patients was relatively small in both cases and in addition to the heterogeneity of the target, no statistically significant modulation of the target was validated [154,155]. To date, no follow-up study has been reported.

**Figure 4.**
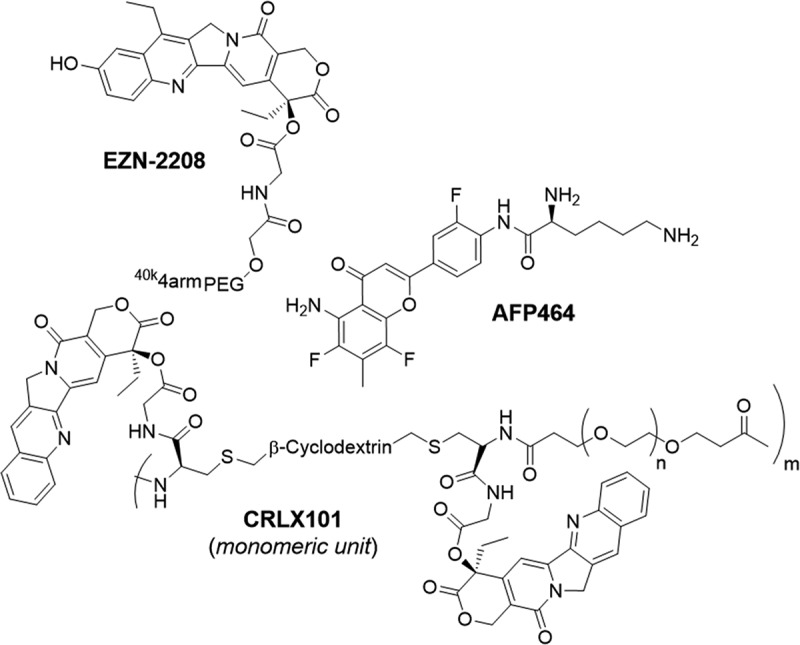
The structures of small molecule-based inhibitors reported to impact HIF-1αexpression and translation.

Topoisomerase 1 inhibitors like Camptothecin and topotecan inhibit HIF-1 induction of luciferase in U2OS HRE-luciferase cells, affecting HIF-1α translation [156]. Among these inhibitors, Inrinotecan (CPT-11) is notable for treating metastatic colorectal cancer, metabolizing into the potent SN-38. However, SN-38’s poor pharmacokinetic properties prompted the development of EZN-2208 (Figure 4), a pegylated SN-38 drug conjugate, demonstrating superior efficacy in downregulating HIF-1α and its gene targets in preclinical studies [157]. Phase 1 and 2 clinical trials of EZN-2208 have shown promising tolerability and activity in advanced malignancies [158–162]. Similarly, Camptothecin (CPT) has been converted into CRLX101 (Figure 4), a nanoparticle-drug conjugate addressing CPT’s solubility issues and toxic metabolites, with preclinical studies demonstrating potent antitumour activity and reductions in HIF-regulated genes *in vivo* [163–165] . However, the efficacy and safety of CRLX101 in clinical trials depend on several factors, including cancer type, treatment protocol, demographics, and dosing regimen [166–170].

Aminoflavone, the active component of the pro-drug AFP464 (Figure 4), was initially identified as an aromatic hydrocarbon receptor (AhR) ligand. Albeit it was later discovered to suppress HIF-1α expression in MCF-7 cells through a mechanism independent of AhR signaling. The exact mode of action of aminoflavone remains unclear, although data suggests it affects mRNA expression and translation of HIF-1α [171].

### Inhibitors of histone deacetylases for HIF-1α destabilisation

The intricate regulation of HIF-1α stabilization mediated by multiple proteins, offers various targets for mitigating HIF-1α stability. Several classes of drugs have been identified to disrupt these proteins and destabilize HIF-1α.

Histone deacetylases (HDACs) function by removing acetyl groups from histones and other proteins. The role of HDACs on HIF-1 stabilization is ambiguous, with reports suggesting stabilizing and destabilizing effects on HIF-1. For instance, HDAC1 is proposed to be involved in the acetylation of Lys-709 on HIF-1α, leading to its destabilization [172]. In contrast, HDAC1 has been demonstrated to directly inhibits the ubiquitination of HIF-1α, thereby stabilizing it, promoting the proliferation of colorectal cancer cells [173]. Evidence suggests that the effect HDAC inhibitors have on the stabilization of HIF-1 vary significantly based on cell type. Despite this, HDAC inhibitors have been investigated for their potential to target cancers driven by hypoxia signaling [173,174].

**Figure 5.**
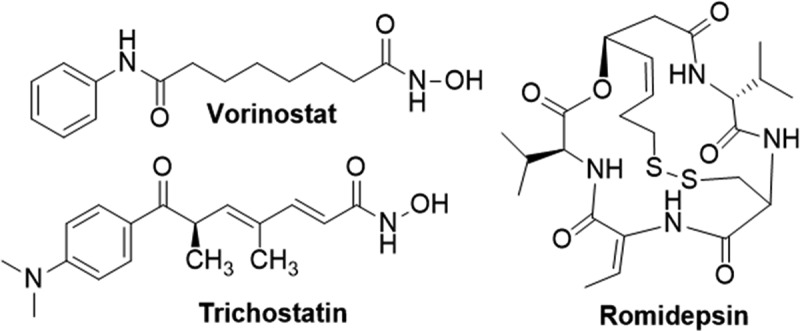
Structures of HDAC inhibitors reported to destabilise HIF-1α.

Vorinostat (Figure 5) is a class I/IIb/IV HDAC inhibitor, FDA approved for the treatment of cutaneous T-cell lymphoma (CTCL) and identified to be a potent inhibitor of HIF-1 signaling. Its mode of action on HIF-1α is complex due to its pan-inhibitory activity on HDACs, with different HDACs reported to exert different effects on HIF-1 mediated signaling. Through inhibition of HDAC1, Vorinostat is proposed to prevent the deacetylation of the HSP90 chaperone, hindering nuclear translocation of HIF-1α [175]. Other studies suggest inhibition of class 2 HDAC activity increases acetylation of HIF-1α, rendering it susceptible to proteasomal degradation mediated by VHL or p53 [61,176]. However, mechanistic studies have failed to demonstrate an increase in VHL activity upon HIF-1α acetylation by N-acetyltransferase [177,178]. Vorinostat has also been proposed to act through HDAC9 to inhibit HIF-1α translation, mediated via interaction with the eukaryotic translation initiation factor – eIF3G [179]. The mechanisms through which Vorinostat impacts hypoxia signaling is intricate and requires further characterization. Despite this complexity, Vorinostat is currently undergoing multiple phase 1 and 2 clinical trials for treating various cancers.

Similar to Vorinostat, Romidepsin (Figure 5) is a natural, depsipeptide inhibitor of class of I/II HDACs, FDA-approved for CTCL treatment. It inhibits HIF-1 activity and notably suppresses VEGF induction, inhibiting tumor angiogenesis in Lewis lung carcinoma model. Romidepsin induces histone acetylation in the P2 region of the VEGF promoter, preventing HIF-1 binding, rather than affecting HIF-1 stability [180]. Another class I/II HDAC inhibitor but not FDA approved for any cancer treatments is Trichostatin (TSA) (Figure 5), a secondary metabolite from *Streptomyces hygroscopius*. It has shown therapeutic potential across various cancers, reducing HIF-1α levels and VEGF expression in human tongue squamous cell carcinoma cells [181,182]. Further exploration of TSA’s mechanism showed that treating PHD2-deficient nucleus pulposus cells with TSA had little impact on HIF-1αlevels, suggesting an influence on the interaction between HIF-1α and PHD2 [174]. The efficacy and safety of TSA in cancer treatment has yet to be evaluated in clinical trials.

### Inhibitors of heat shock protein 90 for HIF-1a destabilisation

HSP90 is an ATP-dependent, molecular chaperone protein found ubiquitously in cells, playing a crucial role in stabilizing various biomolecules, including HIF-1α. Mechanistically, HSP90 competes with RACK1 binding to HIF-1α via interacting with the PAS-B domain, thereby inhibiting the proteasomal degradation of HIF-1α [183]. Several drugs have been identified to inhibit HSP90 and induce HIF-1α degradation.

**Figure 6.**
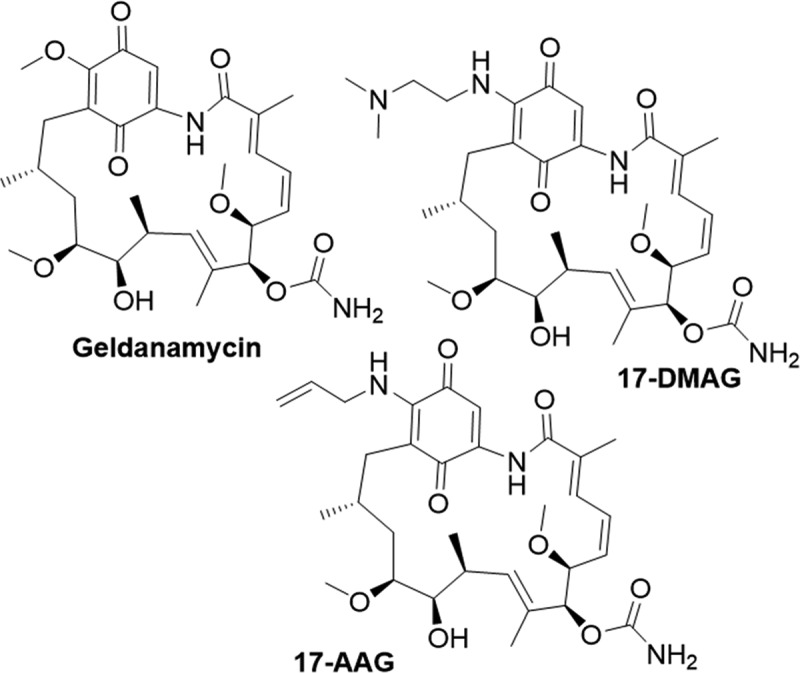
Structures of small molecule inhibitors of HSP90 reported to impact HIF-1α stability.

Geldanamycin (Figure 6), is a natural benzoquinone ansamycin isolated from *Streptomyces hygroscopius* that binds to the ATP-binding site of HSP90 [184]. In prostate cancer PC-3 and LNCaP cells, treatment with geldanamycin induced the degradation of nuclear HIF-1α in a dose- and time-dependent manner via an oxygen independent mechanism. This proteasomal based degradation was confirmed through the use of proteosome inhibitors, which rescued HIF-1α levels [185]. However, Geldanamycin’s clinical utility is limited due to poor physicochemical properties and hepatotoxicity observed in preclinical studies. Derivatives of Geldanamycin, such as 17-allylamino-GDA (17-AAG) (Figure 6) and (17-(2-Dimethylaminoethyl)amino-17-demethoxygeladanamycin (17-DMAG) (Figure 6), have been developed to overcome these unfavorable properties. These derivatives exhibit superior potency and safety profiles and have been shown to destabilize both HIF-1α and HIF-2α, leading to the downregulation of HIF-related genes [186,187]. Of the two, 17-DMAG exhibits the most favorable properties with high antitumour efficacy; however, most Geldanamycin derivatives tested in clinical trials have failed at phase one or two [184,188]. It is worth noting that other classes of HSP90 inhibitors, such as purine, benzamide and resorcinol-based inhibitors, exist; however, as far as we are aware, there are no reports on their effect on HIF-1 or HIF-2.

### Activators of the PHD2/VHL pathway

The stability of HIF-1α is mainly regulated through the actions of PHD2 and VHL. Briefly, PHD2 hydroxylates HIF-1α at two proline residues in the ODD domain, which triggers ubiquitination by VHL-containing E3 ubiquitin ligase and subsequent degradation by the 26S proteasome system. Therefore, drugs that potentially activate the PHDs/VHL/26S proteasome system have been expected to work as HIF-1 inhibitors.

**Figure 7.**
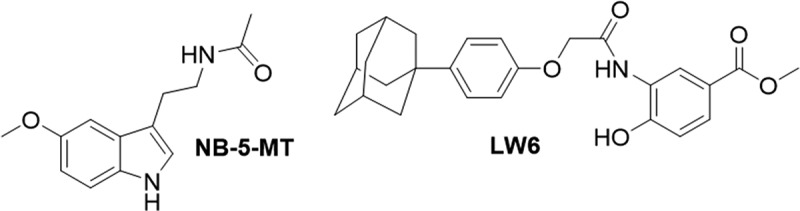
Structures of small molecules the re-activate the PHD2/VHL pathway to induce the proteolysis of HIF-1α.

Derivatives of the hormone Melatonin, known to activate the activity of PHDs have shown success in enhancing the interaction between HIF-1α and the VHL E3 ligase [189,190]. Among these derivatives, *N*-butyryl-5-methoxytryptamine (NB-5-MT) (Figure 7) exhibited the highest anti-tumor potency, resulting in reduced expression of HIF-1α, decreased transcription of HIF-1α gene targets, and inhibition of angiogenesis both *in vitro* and *in vivo*. Additionally, NB-5-MT demonstrated superior pharmacokinetic properties compared to melatonin [191].

An alternative mechanism of promoting proteolysis of HIF-1α via the PHD2/VHL pathway involves increasing the expression of the VHL E3 ligase. An aryloxyacetylamino benzoic acid compound was identified from a high-throughput cell-based HRE reporter screen in Hep3B cells and after SAR studies identified LW6 (Figure 7) [192]. It was discovered that LW6 accelerated the proteasomal degradation of HIF-1α WT by increasing VHL expression, demonstrating excellent potency *in vivo* in mice with HCT116 ×enografts [193]. Further investigations revealed rapid conversion of LW6 into (4-adamantan-1-yl-phenoxy)acetic acid (APA), an active metabolite contributing to its anti-tumor activity [192]. However, the pharmacokinetic properties of LW6 were unfavorable due to its short half-life and poor bioavailability, necessitating further lead optimization before submission to clinical trials [194]. Despite these challenges, the efficacy demonstrated in tumor models and low toxicity suggest potential for further development of this novel inhibitor type.

### Inhibition of co-activator recruitment (p300/CBP)

To initiate transcription, the CTAD domain of HIF-1 binds to the cysteine histidine-rich domain 1 (CH1) of p300/TAZ1 (transcription adaptor zinc-binding domain) domain of CBP. Overexpression of HIF-1α CTAD polypeptides was found to actively compete with HIF-1α for the CBP binding site, leading to the disruption of their interaction, and consequently impairing the transcriptional activity of HIF-1 [195]. Inhibiting this interaction between HIF-1α and its co-factors has been the focus of extensive research with numerous inhibitors reported.

**Figure 8.**
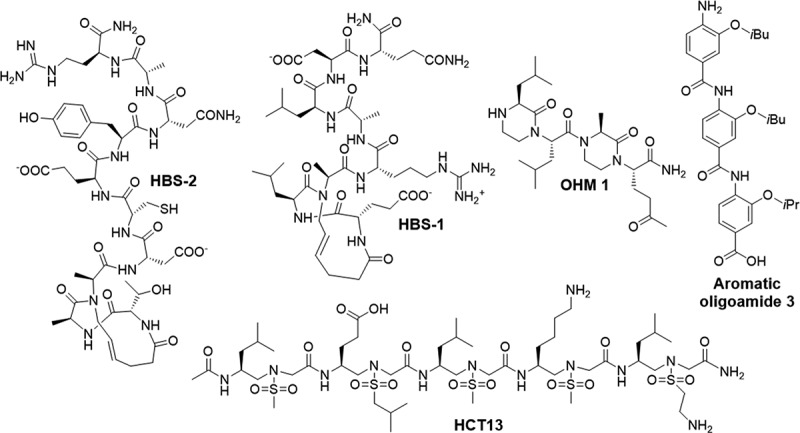
Structures of proteomimetic inhibitors of the p300 and CBP co-activators.

The interface between p300/CBP and the C-TAD domain of HIF-1α is vast, covering an area of 3393 A^2^. The C-TAD domain is inherently disordered in its unbound state, posing challenges for direct targeting of HIF-1α via this strategy. However, upon binding with p300/CBP, the C-TAD domain undergoes a conformational change characterized by the formation of 3 helices that wrap around the TAZ1 domain. Mutagenesis studies have demonstrated the crucial role of these helices in the binding process. Deletion of key residues with helices B (Cys-800, Asn-803) and C (Leu-818, Leu-822) lead to a loss in binding [43,196]. With these key structural insights, efforts have been made to design inhibitors that mimic the essential binding interactions of the HIF-1α helices. This includes a stabilized α-helical peptide design utilizing hydrogen bond surrogates (HBS) to mimic the DCEVNA sequence of helix B, which contains, Cys-800 and Asn-803, critical residues for binding. By replacing the N-terminal main chain hydrogen bond with a carbon–carbon bond via ring closing metathesis, they synthesized a series of HBS to replicate the α-turns of the native peptide, confirmed by circular dichroism spectroscopy (CDS) to closely resemble helix-C. These peptides bound to p300, with the most potent peptide (HSB2) (Figure 8) achieving a K_D_ = 420 nM as determined by ITC. Although initial cell experiments showed limited activity due to poor cell permeability the addition of an arginine at the C-terminal significantly enhanced cellular uptake and peptide activity. Moreover, the HBSs peptides down-regulated hypoxia-induced transcription of VEGF genes in Hela cells [197]. A follow-up study on designing mimetics of helix C and identified Leu-822, Asp-823 and Gln-824 as critical residues on HIF-1α C-TAD for binding p300/CBP through computational alanine scanning. Using the same HBS peptide strategy, they prepared a ELARALDQ HBS sequence which exhibited helical conformations akin to the native peptide. The most potent inhibitor, HBS-1 (Figure 8) achieved a K_D_ = 690 nM and effectively inhibited hypoxia-induced transcription in cells, confirmed through luciferase-based reporter assays and q-PCR. Further assessment *in vivo* with mice bearing RCC xenograft tumors demonstrated a reduction in tumor volumes by over 50% compared to control groups [198]. The group also described another class of a helix mimetics utilizing oxopiperazines, which are generated by bridging ethylene bridges between adjacent amides, affording nonpeptide chiral scaffolds with protein-like functionality. A series of oxopiperazines, has been reported, with one compound called OHM-1 (Figure 8) designed to mimic the helix C of the C-TAD domain, bearing key binding residues Leu-818, Leu-822 and Gln-824. OHM-1 exhibited strong binding affinity to p300 and inhibited HIF-1 dependent transcription in a dose-dependent manner. *In vivo* studies conducted in mice with MDA-MB-231 ×enografts of triple-negative breast cancer cells revealed a median reduction in tumor volume of roughly 50% with no significant signs of toxicity detected [199].

An unnatural helical sulfonyl- γ-AApeptide foldamers has also been reported to mimic the α-helical structure of helix C. Computational modeling resulted in sulfonyl- γ-AApeptides harboring achiral sulfonyl groups with side chains mimicking residues Leu-818-Leu-822-Val-825, crucial for binding. Among these designs, HC14 demonstrated the most precise alignment of sulfonyl side-chains with key helix C residues. CD studies confirmed the helical conformation of these sulfonyl- γ-AApeptides, and protein binding assays including fluorescence polarization (FP) and microscale thermophoresis (MST) confirmed binding affinity to p300. Experiments in HeLa cells confirmed the lead molecule’s (HC13) (Figure 8) ability to inhibit hypoxia-induced gene transcription. Additionally, these peptides displayed high cellular permeability and favorable stability and pharmacokinetic properties [200].

In attempt to obtain small molecule proteomimetic inhibitors of this interaction, aromatic oligoamides were designed to project side chains of helix C that are essential for PPI between C-TAD of HIF-1α and p300. Using a 3-*O*-alkylated oligobenzamide scaffold, molecules were designed that recapitulate the Leu-141-Leu-145-Val-148 sidechain interactions and bind p300. Aromatic oligoamide 3 (OHM 3) (Figure 8) was identified to be the most potent binder to the CH1 domain of p300 with an IC_50_ of 9.19 μM. SAR revealed that scope for diversification on these sidechains were limited with aromatic, shorter alkyl and polar sidechain derivatives being detrimental to binding affinity [201].

**Figure 9.**
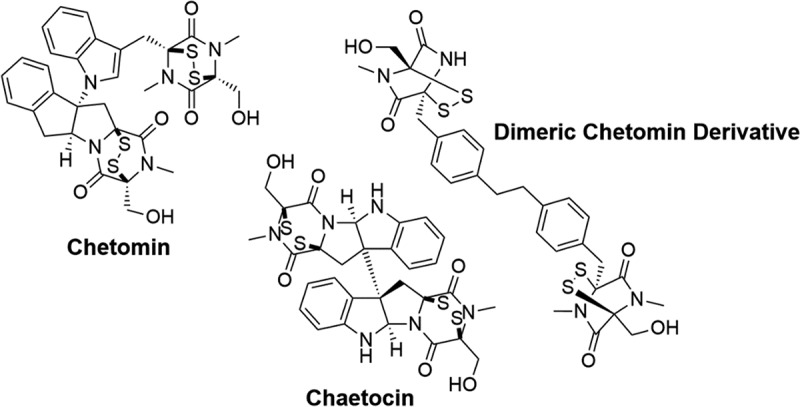
Structures of small molecules that induce the ejection of Zn(II) ions from the p300 co-activator.

An alternative approach involves targeting the zinc-binding domain, which was discovered serendipitously during a screen aimed to identify inhibitors of the HIF-1α/p300 interaction. Chetomin (Figure 9), an epidithiodiketopiperzine (ETP) isolated from fungi was identified with an inhibitory activity >50% in their ELISA high-throughput screen assay. Chetomin interfered with hypoxia-induced transcription and demonstrated excellent antitumour efficacy *in vivo*. Notably, Chetomin possesses a unique disulfide link that imposes significant torsional strain, resulting in an unconventional conformation. Due to this peculiar structure, Chetomin’s mode of action was further investigated; it was found that the electrophilic functionality of the ETP core caused the ejection of the Zn(II) ion in the CH1 domain of p300, thereby disrupting the tertiary structure of CH1 and preventing binding to HIF-1α [202]. This work prompted further studies aiming to enhance the ETP scaffold; including developing dimeric ETP molecules (Figure 9) with markedly improved activity compared to Chetomin [203]. However, the clinical potential of the ETP scaffold is limited due to the local toxicity identified in mouse studies. In pursuit of molecules with therapeutic potential, a similar high through-put screen was performed using natural products; indandione and benzoquinone derivatives were identified to induce Zn(II) ejection through a similar electrophilic-promoted mechanism [204].

### Activators of FIH

**Figure 10.**
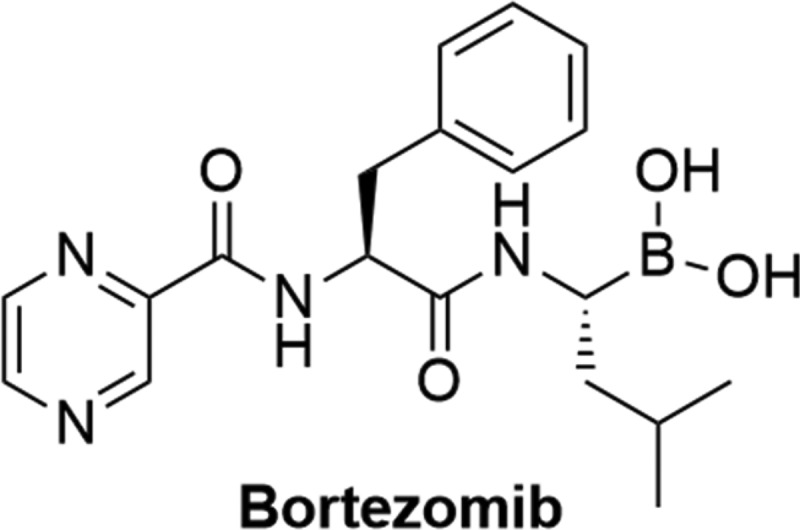
The structure of bortezomib, a proteasome inhibitor reported to promote the interaction between HIF-1α and FIH.

Another approach involves indirectly targeting the interaction between p300/CBP and HIF-1α by targeting proteins involved in regulating their interactions. FIH hydroxylates HIF-1α on Asn803, effectively inhibiting the binding between HIF-1a and its co-activators [64]. Bortezomib (Figure 10), an FDA approved proteasome inhibitor utilized in the treatment of multiple myeloma (MM) and mantle-cell lymphoma, modulates the balance of pro-apoptotic and anti-apoptotic proteins [205–207]. When examining Bortezomib’s antitumour effects in MM, researchers observed an indirect suppression of angiogenesis through decreased VEGF levels and a reduction in hypoxia adaptation, indicated by diminished CAIX expression [205,206,208]. It has been demonstrated that Bortezomib attenuated the hypoxic induction of erythropoietin and VEGF at sub nanomolar concentrations in MM and liver cancer by promoting the interaction between the C-TAD domain of HIF-1α and FIH [209]. Notably, Bortezomib has demonstrated minimal impact on HIF-2 mediated signaling. However, the molecular basis underlying Bortezomib’s selectively between the two isoforms remains to be fully elucidated [210].

### Inhibition of DNA binding

**Figure 11.**
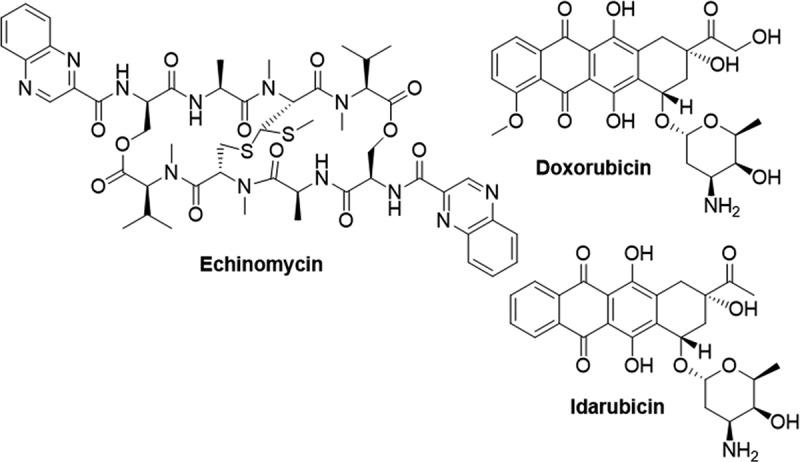
Structures of molecules capable of preventing HIF-1 from binding to the HRE promoter.

Given the necessity of HIF-1’s binding to the HRE promoter region for transcription activation, disrupting this interaction has become another strategy to inhibit hypoxia-mediated transcription. Echinomycin (Figure 11) was identified through a high-throughput ELISA screen aimed for inhibitors of HIF-1 DNA binding. Chromatin immunoprecipitation (ChIP) experiments revealed that Echinomycin selectively intercalated into the HRE promoter of VEGF, thereby inhibiting HIF-1 binding. Moreover, Echinomycin demonstrated inhibition of HIdF-1 dependent luciferase in U251-HRE cells and suppressed the hypoxic induction of VEGF expression in U251 cells [211]. Anthracyclines have also been reported to inhibit hypoxia signaling in cancer cells by the same mechanism. For example, doxorubicin (Figure 11) has been shown to disrupt hypoxia response, suppressing migration of RCC cells, and impairing hypoxia-mediated angiogenic responses in mice carrying HeLA xenografts [212]. Idarubicin (IDA, Figure 11) was shown to inhibit hypoxia signaling in pheochromocytomas (PHEO) by blocking the binding of HIF-1 and HIF-2 to the HRE sequence. IDA was found to significantly repress growth of Hep3B cells and *in vivo* tumor formation in mice models of PHEO [213].

### Discovery of direct inhibitors and clinical candidates of HIF-2α/HIF-1β

Designing molecules that directly target HIF-α subunits has proven challenging due to the absence of conventional druggable pockets. However, the PAS domains in these subunits play a crucial role in stabilizing the HIF heterodimer by mediating PPIs between α and βsubunits [36,37]. While PAS domains in other proteins respond to changes in internally bound co-factors, such as small molecules, HIF proteins lack such cofactors [214]. Protein crystallography identified a potential binding cavity within the HIF-2α PAS-B domain, prompting an NMR-based ligand screen which identified binders to the domain. This led to the development of THS-044, which was found to disrupt PAS B heterodimerisation and inhibited HIF-2 function in cells. Notably, these compounds did not bind to the corresponding HIF-1α PAS-B domain despite sequence homology [215]. Further screening led to the identification of Benzoxadizole 1, with subsequent modifications resulting in Benzoxadizole 2, which effectively disrupted HIF-2 DNA binding and transcription of target genes *in vitro*. However, limited *in vivo* activity was observed, attributed to interference from the conformationally flexible Met-252 side chain within the HIF-2α PAS-B domain [216,217].

In a subsequent study, the same research group investigated a tetrazole inhibitor series, which featured compounds with two chiral centers, differing from the achiral benzoxadizole leads. They aimed to enhance the conformational flexibility of their lead compounds by employing a chiral scaffold, thereby exploring underutilized regions within the HIF-2α PAS-B domain cavity. Unlike the benzoxadizole compounds, the tetrazole hits lacked a metabolically unfavorable nitro group crucial for the activity of the benzoxadizole series [218]. Enantiomer studies revealed that the 2S, 4R configuration of the aromatic rings exhibited superior activity compared to the enantiomers with the 2R, 4S configuration. The most active compounds, Tetrazole 37, showed a K_D_ of 23 nM by ITC and an IC_50_ of 43 nM in a luciferase proximity assay. However, no subsequent studies have been disclosed regarding this tetrazole series of inhibitors, possibly due to the low hydrophilicity of the lead compound, limiting its oral bioavailability for clinical use [219].

**Figure 12.**
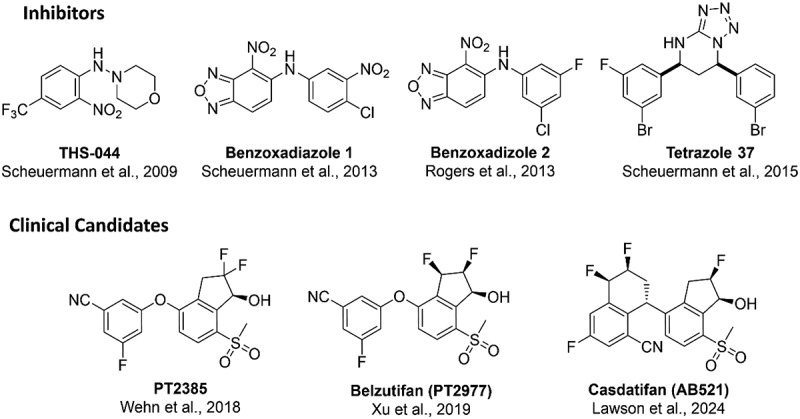
Structures of small molecules inhibitors and clinical candidates of HIF-2α.

The benzoxadiazole inhibitor series, identified as the most promising lead, underwent structural optimization that revealed the polarized nitrobenzoxadiazole moiety crucially enhanced an n → p*_Ar_ interaction with Tyr-281’s phenolic oxygen (based on co-crystallization analysis with benzoxadizole 2 and the HIF-2α PAS-B domain). This hypothesis was validated by substituting the oxadiazole fragment and nitro group with stronger electron withdrawing groups, enhancing activity by reducing electron density in the aromatic ring. Further potency improvements were explored through modifications at the 3-position of the ring, leveraging the flexibility conferred by nearby side chains of His-292 and Met-252. Addition of a methylol group, significantly increased activity, with structural analysis revealing hydrogen bond formation with His-293, Tyr-281, and a water molecule. Further efforts focused on rigidifying the hydroxyl group conformation to restrict its flexibility, resulting in two series of compounds featuring cyclic secondary alcohols, including cyclic sulfone and indanol analogues. Among these, the S-configuration of the hydroxyl group in the indanol analogues exhibited notably improved activity compared to its enantiomer counterpart. Additionally, installing a geminal difluoro electron-withdrawing group further improved potency. Following extensive pharmacokinetic/pharmacodynamic (PK/PD) studies on selected analogues, PT2385 (Figure 12) emerged as the lead candidate due to its optimal balance of drug-like properties and potency, thus advancing it to a phase 1 clinical trial [220].

The Phase 1 clinical trial of PT2385 involved dose escalation studies with patients diagnosed with heavily pretreated ccRCC. Despite significant variability in drug exposure among patients, PT2385 exhibited a favorable safety profile and potent efficacy [221]. Further PK investigations revealed that variations in PT2385 exposure were due to direct glucuronidation at the hydroxyl group, catalyzed by the uridine 5’-diphosphoglucuronosyltransferease 2B17 (UGT2B17). This metabolic pathway rationalizes the findings from dose escalation studies, where doses exceeding 800 mg failed to increase PT2385 exposure significantly due to UGT2B17 metabolism. Pronounced glucuronidation was attributed to the strong inductive effects of the geminal difluoro group, increasing the reactivity of the hydroxyl group. Optimisation studies aimed to mitigate glucuronidation while preserving or improving potency. This involved relocating the trans fluorine group to a cis configuration at position 3 on the indanol, maintaining favorable electron withdrawing properties on the aromatic ring. The resulting compound (named PT2977) (Figure 12) exhibited reduced glucuronide formation rates compared to PT2385 in addition to enhanced potency. Preclinical PK/PD studies revealed superior metabolic stability and *in vivo* activity of PT2977 compared to PT2385 [222]. Subsequent clinical trials demonstrated favorable safety and anti-tumor activity of PT2977 as a monotherapy for patients with advanced or metastatic ccRCC [223–225]. Following the acquisition of Peloton therapeutics by Merck, PT2977 was renamed Belzutifan. Following a phase 2 clinical trial, the FDA approved Belzutifan as a first-in-class HIF-2 inhibitor for adult patients with VHL disease associated tumors in 2021 [226]. The success of Belzutifan led Arcus Biosciences to further explore its potential. Resulting in the development of AB251, later named Casdatifan (Figure 12). As a derivative of Belzutifan, Casdatifan demonstrates enhanced potency along with improved PK and PD profiles. It is currently under investigation in phase 1 clinical trials for the treatment of RCC [227].

### Discovery of direct inhibitors of HIF-1α/HIF-1β

The multiple bulkier side chains within the HIF-1α PAS-B cavity (c.f. that of HIF-2α) significantly reduce the size of the pocket [216]. Consequently, alternative strategies have been pursued to develop inhibitors that inhibit HIF-1 activity by directly engaging with HIF-1α.

**Figure 13.**
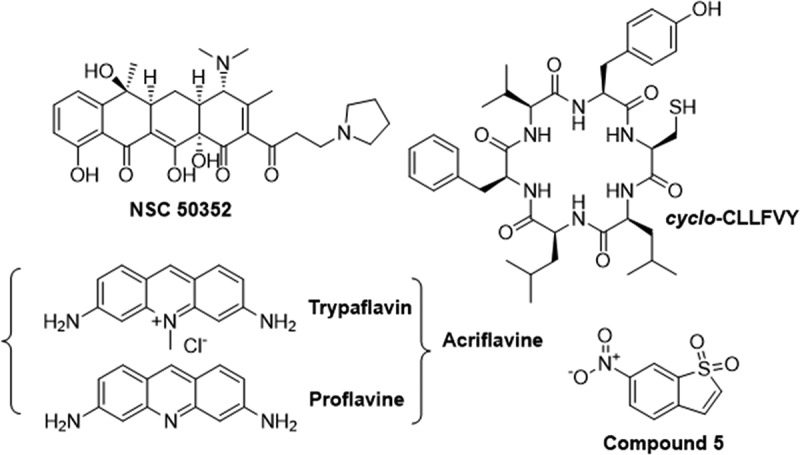
Structures of inhibitors reported to directly bind to HIF-1α.

Initial efforts to directly target HIF-1 focused on the PAS-A domain of HIF-1α, with a high throughput ELISA screen using FLAG-HIF-1β-PAS-A and HIF-1α-PAS-A-His with chemical libraries from the National Cancer Institute (NCI). This screen identified NSC 50,352, (Figure 13) a rolitetracycline compound, which exhibited concentration-dependent inhibition of the interaction with an IC_50_ of 1.4 μM. However, limited activity was observed in cell-based assays, likely attributed to poor membrane permeability or an inability to inhibit the endogenous interaction between HIF-1α and HIF-1β [228].To date, no further optimization of this molecule has been reported.

Another HTS study, identified Acriflavine (ACF) (Figure 13), an FDA approved antimicrobial existing as a mixture of trypaflavine and proflavine, inhibits HIF-1 heterodimerisation by targeting the PAS-B domains of both HIF-1α and HIF-2α. *In vitro* studies showed ACF’s dose-dependent inhibition of HIF-1 DNA binding and transcriptional activity in cancer cells. In mice with prostate cancer xenografts, ACF prevented tumor vascularization and expression of angiogenic cytokines, halting tumor growth [229]. The authors questioned whether if it was plausible for ACF to occupy the same PAS-B binding site as the benzoxadiazole series of HIF-2α inhibitors. Crystallisation studies conducted on the HIF-2 dimer in complex with ACF revealed a unique mode of action, with ACF intercalating into an interfacial pocket formed by HIF-2α PAS-B and the HIF-1β PAS-A domains. Further structural analysis revealed that amino acid residues Arg-266 and Val-305 of HIF-1β made direct contact with ACF destabilizing the interface. Mutagenesis studies demonstrated the critical roles of these residues in maintaining the integrity of one of four interfaces that stabilize the HIF-α-HIF-1β heterodimer. Binding experiments with both HIF-1 and HIF-2 validated these findings, with ACF exhibiting a binding affinity of approximately 40 nM for both heterodimers [230]. On going research explores ACF’s therapeutic potential across various cancers [231–241]. ACF has also demonstrated efficacy in combination-based therapies enhancing drug-uptake, overcoming drug resistance, and improving survival rates [233,238,242–244]. Despite promising preclinical results, no clinical trials have evaluated its efficacy against HIF-driven cancers. One challenge associated with ACF is its lack of selectivity [231]. ACF has been reported to interact with not only HIFs but also protein kinases, topoisomerase 1 and 2 and is well-known for its ability to intercalate between DNA strands [231,234,245]. It should also be noted that the original manuscript reporting ACF as an inhibitor of HIF-1 and HIF-2 has now been withdrawn due to data manipulation.

Covalent inhibitors targeting HIF-1α’s PAS-B subdomain has also been explored. To enable structural studies, a HIF-1α R245E and HIF-1β E362R mutant were used. Analysis revealed an internal cavity within the PAS-B subdomain of HIF-1α containing two cysteine residues, Cys-255 and Cys-377, as potential targets. Screening 170 electrophile fragments, they identified five compounds capable of modulating Cys-255. Compound 5 (Figure 13) selectively bound to Cys-255 with an IC_50_ of 43 nM and NMR analysis confirmed a change in conformation of the HIF-1α’s PAS-B domain. Further biophysical characterization via AlphaScreen and Surface Plasmon Resonance (SPR) indicated that compound 5 reduced the affinity of HIF-1α PAS-B for HIF-1β PAS-B by 10-fold [246]. However, no data was provided regarding the compound’s ability to inhibit HIF-1 mediated signaling in cancer cells, likely due to selectivity of the highly electrophilic vinyl sulfone which could promiscuously label exposed cysteine residues on other proteins.

Peptides have emerged as effective inhibitors of HIF interactions, exemplified by cyclic hexapeptide *cyclo*-CLLFVY (Figure 13), which was discovered through a Split Intein Circular Ligation of Peptides and Protein (SICLOPPS) screen targeting the HIF-1 transcription factor. This screening method uses an intracellular library of 3.2 million cyclic hexapeptides (*cyclo*-CXXXXX) in combination with an *Escherichia coli (E. coli)* cells HIF-1 reverse-two hybrid system. Conjugated with a releasable cell-penetrating peptide tag, Transactivator of Transcription (TAT) peptide, *Cyclo-*C(TAT)LLFVY demonstrated selective binding to the PAS-B domain of HIF-1α, exhibiting a K_D_ of 124 nM by ITC and effectively inhibited HIF-1 signaling in hypoxic MCF-7 and U2OS cancer cells. Notably, *cyclo*-CLLFVY displayed isoform specificity, with no discernible binding or impact on the function of the HIF-2α isoform [247]. In a following study, human HEK292-cells were genetically modified to conditionally express the split-intein that synthesizes of *cyclo-*CLLFVY *in vitro* via splicing chemistry upon exposure to a hypoxic environment. Subsequent experiments validated the inhibition of HIF-1 dimerization and hypoxia-response signaling in these engineered cells [248]. There is currently on-going work in our laboratory to optimize the activity of this cyclic peptide.

### Discovery of dual inhibitors of both hypoxia inducible factor 1 & 2

**Figure 14.**
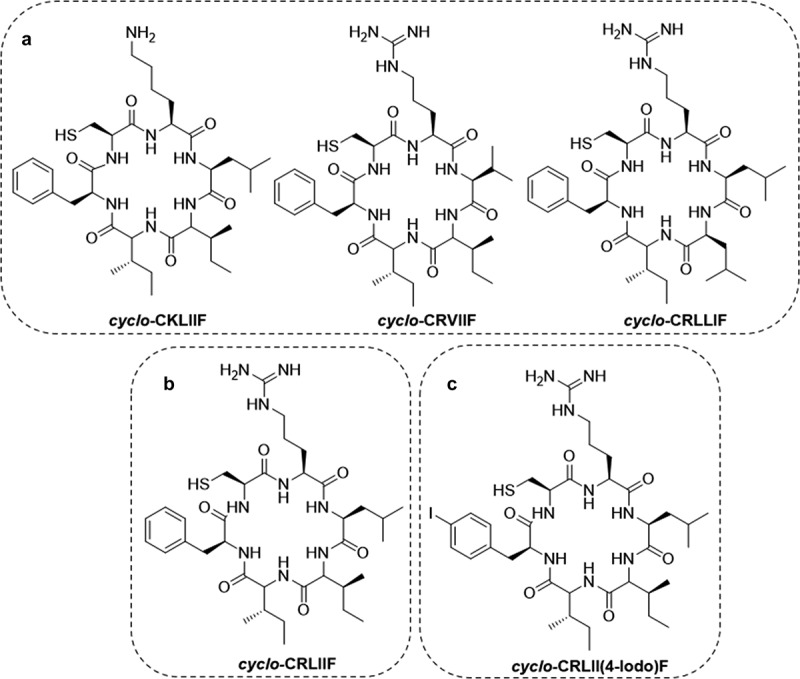
Cyclic peptide inhibitors of both HIF-1 and HIF-2. (a) cyclic peptides pulled from SICLOPPS screening that bind the PAS-B domain of both HIF-1α and HIF-2α. (b) cyclic peptide inhibitor designed from hits pulled from the SICLOPPS screen. (c) the most potent cyclic peptide inhibitor optimised from *cyclo*-CRLIIF through SAR studies.

Given the prevalent elevation of both HIF-1α and 2α levels in multiple cancers and their direct correlation with this and poor patient outcome, there is significant potential for dual inhibitors targeting both HIF-1α and HIF-2α activity. The SICLOPPS screening platform was again used for this purpose combining a 3.2 × 10^6^ *cyclo*-CXXXXX cyclic peptide library, with an *E. coli* RTHS reporting on the HIF-2α/HIF1β PPI and the previously mentioned HIF-1 RTHS. In this assay, colonies that survived on selective media potentially contain inhibitors of this PPI. The SICLOPPS plasmids were isolated from surviving colonies and transformed into the HIF-1 RTHS. Following verification of hits, 3 cyclic peptides (*cyclo*-CKLIIF, *cyclo*-CRVIIF and *cyclo*-CRLLIF) (Figure 14) were identified, conferring significant growth advantages to HIF-1 RTHS containing *E*. *coli* on selective media. Subsequent MST binding assays, using the PAS-B domains of both alpha isoforms identified *cyclo*-CRLIIF (Figure 14) as the lead inhibitor for further optimization. Despite not being an isolated sequence from SICLOPPs, they found performing a L4I switch on *cyclo*-CRLLIF to reflect the other 2 hits gave a ~ 3-fold improvement in K_D_. Computational docking studies were performed with this cyclic peptide on both PAS-B domains, revealing that the peptide binds to the same site as a crucial “hotloop”, from HIF-1β, offering a plausible inhibition mechanism between the α and β subunits. SAR-guided studies were conducted on *cyclo-*CRLIIF through alanine scanning, MST, and incorporation of non-natural amino acids. Among the derivatives, *cyclo*-CRLII (4-iodo)F (Figure 14) emerged as the most potent cyclic peptide binder. *Cyclo*-CRLII(4-iodo)F effectively inhibited hypoxia-response signaling of both HIF isoforms across various cancer cell lines, including 786-O, a ccRCC cell line predominately driven by HIF-2 [249]. Collectively, computational and cell-based data suggest a unique mechanism of action of *cyclo*-CRLII(4-iodo)F compared to the selective HIF-2 inhibitor Belzutifan, positioning the cyclic peptide as a promising candidate in the development of a dual HIF inhibitor for cancer therapy.

## Conclusion and future perspective

Solid tumors contribute significantly to global mortality rates. The hypoxic core and microenvironments of these tumors create harsh conditions that triggers the homeostatic cellular response regulated by HIFs. Activation of the HIF pathway leads to the transcription of hundreds of genes, crucial for cellular adaptation, survival, and growth in hypoxia contributing to the development of more aggressive cancer phenotypes, resulting in poor patient prognosis. Understanding the complex mechanism of HIFs has been vital for devising strategies to combat this transcription factor in tumors. Nonetheless, directly targeting HIFs through conventional drug discovery methods poses challenges. HIFs facilitate their function through PPIs, a target sub-class that presents inherent difficulties for conventional small molecule intervention. Despite this obstacle, there are reasons for optimism as a considerable number of compounds, including approved drugs, have demonstrated inhibitory effects of the hypoxia mediated pathway. Many of the approaches undertaken thus far have focused on indirect targets, such as HIF mRNA expression, protein synthesis, HIF stabilizers, HIF co-activators and DNA binding. Many of these agents were originally developed as clinical candidates to target other cancer-associated proteins, with their impact on HIFs being a serendipitous discovery. Advances have also been made in inhibiting co-activator recruitment using peptidomimetic compounds. Another promising avenue involves the direct targeting of HIFs with notable progress observed in this field, especially in the development of HIF-2 inhibitors like Belzutifan, used in the treatment of advanced or metastatic ccRCC. However, Belzutifan demonstrated modest efficacy against RCC in clinical trials with 51% of patients not exhibiting tumor regression, 3% showing progressive disease, and partial response in the remaining patient cohort. Inactivating mutations, such as the known G323E mutation in HIF-2α PAS-B and F446L in HIF-1β, may be to blame, necessitating the development of direct HIF inhibitors with different binding sites [226]. Another hypothesis posits that the hypoxia response in this population’s tumors is driven by both HIF-1 and HIF-2, suggesting potential benefits from combinatorial therapy involving inhibitors targeting both HIF-1 and HIF-2, or the use of a dual inhibitor. Several reviews have underscored the necessity for a dual inhibitor, particularly given the transition that many cancers undergo from HIF-1 to HIF-2 as hypoxic conditions progress from acute to chronic states in solid tumors. The recent discovery of cyclic peptides capable of binding to both isoforms, coupled with an increased understanding of the structures and molecular mechanisms of HIFs, holds promise for the development of novel inhibitors and clinical candidates directly targeting HIFs.

## References

[1] Semenza GL. Hypoxia-inducible factors in physiology and medicine. Cell. 2012;148(3):399–408. doi: 10.1016/j.cell.2012.01.021 22304911 PMC3437543

[2] Vannucci RC, Brucklacher RM, Vannucci SJ. Glycolysis and perinatal hypoxic-ischemic brain damage. Dev Neurosci. 2005;27(2–4):185–190. doi: 10.1159/000085991 16046853

[3] Semenza GL. Regulation of oxygen homeostasis by hypoxia-inducible factor 1. Physiology. 2009;24(2):97–106. doi: 10.1152/physiol.00045.2008 19364912

[4] Wenger RH. Cellular adaptation to hypoxia: O 2-sensing protein hydroxylases, hypoxia-inducible transcription factors, and O 2-regulated gene expression. Faseb J. 2002;16(10):1151–1162. doi: 10.1096/fj.01-0944rev 12153983

[5] Kierans SJ, Taylor CT. Regulation of glycolysis by the hypoxia-inducible factor (HIF): implications for cellular physiology. J Physiol. 2021;599(1):23–37. doi: 10.1113/JP280572 33006160

[6] Zimna A, Kurpisz M. Hypoxia-inducible factor-1 in physiological and pathophysiological angiogenesis: applications and therapies. Biomed Res Int. 2015;2015:1–13. doi: 10.1155/2015/549412 PMC4471260 26146622

[7] Haase VH. Regulation of erythropoiesis by hypoxia-inducible factors. Blood Rev. 2013;27(1):41. doi: 10.1016/j.blre.2012.12.003 23291219 PMC3731139

[8] Hayashi M, Yoo YG, Christensen J, et al. Cell cycle requirement of evading apoptosis for HIF-1α-induced malignant progression in mouse cells. Cell Cycle. 2011;10(14):2364–2372. doi: 10.4161/cc.10.14.16313 21654209 PMC3322472

[9] Chen Y, Gaber T. Hypoxia/Hif modulates immune responses. Biomedicines. 2021;9(3):260. doi: 10.3390/biomedicines9030260 33808042 PMC8000289

[10] Jun JC, Rathore A, Younas H, Gilkes D, Polotsky VY. Hypoxia-inducible factors and cancer. Curr Sleep Med Rep. 2017;3(1):1–10. doi: 10.1007/s40675-017-0062-7 28944164 PMC5607450

[11] Semenza GL. Targeting HIF-1 for cancer therapy. Nat Rev Cancer. 2003;3(10):721–732. doi: 10.1038/nrc1187 13130303

[12] Ledford H, Callaway E. Biologists who decoded how cells sense oxygen win medicine Nobel. Nature. 2019;574(7777):161–162. doi: 10.1038/d41586-019-02963-0 31595071

[13] Karpinets T, Foy B. Tumorigenesis: the adaptation of mammalian cells to sustained stress environment by epigenetic alterations and succeeding matched mutations. Carcinogenesis. 2005;26(8):1323–1334. doi: 10.1093/carcin/bgi079 15802302

[14] Walsh JC, Lebedev A, Aten E, Madsen K, Marciano L, Kolb HC. The clinical importance of assessing tumor hypoxia: relationship of tumor hypoxia to prognosis and therapeutic. Antioxid Redox Signal. 2015;22(10):878–880. doi: 10.1089/ars.2014.6155 25340660

[15] Vaupel P, Harrison L. Tumor hypoxia: causative factors, compensatory mechanisms, and cellular response. Oncologist. 2004;9(S5):4–9. doi: 10.1634/theoncologist.9-90005-4 15591417

[16] Li Y, Zhao L, Li XF. Hypoxia and the tumor microenvironment. Technol Cancer Res Treat. 2021;20:20. doi: 10.1177/15330338211036304 PMC8358492 34350796

[17] Chaudary N, Hill RP. Hypoxia and metastasis. Clin Cancer Res. 2007;13(7):1947–1949. doi: 10.1158/1078-0432.CCR-06-2971 17404073

[18] Eales KL, Hollinshead K, Tennant DA. Hypoxia and metabolic adaptation of cancer cells. Oncogenesis. 2016;5(1):e190–e190. doi: 10.1038/oncsis.2015.50 26807645 PMC4728679

[19] Harris AL. Hypoxia — a key regulatory factor in tumour growth. Nat Rev Cancer. 2002;2(1):38–47. doi: 10.1038/nrc704 11902584

[20] Teicher BA. Hypoxia and drug resistance. Cancer Metast Rev. 1994;13(2):139–168. doi: 10.1007/BF00689633 7923547

[21] Codony VL, Tavassoli M. Hypoxia-induced therapy resistance: available hypoxia-targeting strategies and current advances in head and neck cancer. Transl Oncol. 2021;14(3):101017. doi: 10.1016/j.tranon.2021.101017 33465746 PMC7814189

[22] Vaupel P. Hypoxia and aggressive tumor phenotype: implications for therapy and prognosis. Oncologist. 2008;13(S3):21–26. doi: 10.1634/theoncologist.13-S3-21 18458121

[23] Semenza GL. Pharmacologic targeting of hypoxia-inducible factors. Annu Rev Pharmacol Toxicol. 2019;59(1):379–403. doi: 10.1146/annurev-pharmtox-010818-021637 30625281

[24] Semenza GL, Nejfelt MK, Chi SM, Antonarakis SE. Hypoxia-inducible nuclear factors bind to an enhancer element located 3’ to the human erythropoietin gene. Proc Natl Acad Sci USA. 1991;88(13):5680–5684. doi: 10.1073/pnas.88.13.5680 2062846 PMC51941

[25] Semenza GL, Wang GL. A nuclear factor induced by hypoxia via de novo protein synthesis binds to the human erythropoietin gene enhancer at a site required for transcriptional activation. Mol Cell Biol. 1992;12(12):5447–5454. doi: 10.1128/MCB.12.12.5447 1448077 PMC360482

[26] Maxwell PH, Pugh CW, Ratcliffe PJ. Inducible operation of the erythropoietin 3’ enhancer in multiple cell lines: evidence for a widespread oxygen-sensing mechanism. Proc Natl Acad Sci U S A. 1993;90(6):2423. doi: 10.1073/pnas.90.6.2423 8460154 PMC46099

[27] Firth JD, Ebert BL, Pugh CW, et al. Oxygen-regulated control elements in the phosphoglycerate kinase 1 and lactate dehydrogenase a genes: similarities with the erythropoietin 3’ enhancer. Proc Natl Acad Sci USA. 1994;91(14):6496–6500. doi: 10.1073/pnas.91.14.6496 8022811 PMC44229

[28] Wang GL, Semenza GL. Purification and characterization of hypoxia-inducible factor 1. J Biol Chem. 1995;270(3):1230–1237. doi: 10.1074/jbc.270.3.1230 7836384

[29] Reyes H, Reisz-Porszasz S, Hankinson O. Identification of the ah receptor nuclear translocator protein (arnt) as a component of the DNA binding form of the ah receptor. Sci. 1992;256(5060):1193–1195. doi: 10.1126/science.256.5060.1193 1317062

[30] Ema M, Taya S, Yokotani N, et al. A novel bHLH-pas factor with close sequence similarity to hypoxia-inducible factor 1α regulates the VEGF expression and is potentially involved in lung and vascular development. Proc Natl Acad Sci U S A. 1997;94(9):4273. doi: 10.1073/pnas.94.9.4273 9113979 PMC20712

[31] Tian H, Mcknight SL, Russell DW. Endothelial PAS domain protein 1 (EPAS1), a transcription factor selectively expressed in endothelial cells. Genes Dev. 1997;11(1):72–82. doi: 10.1101/gad.11.1.72 9000051

[32] Hogenesch JB, Chan WK, Jackiw VH, et al. Characterization of a subset of the basic-helix-loop-helix-pas superfamily that interacts with components of the dioxin signalling pathway. Journal Of Biological Chemistry. 1997;272(13):8581–8593. doi: 10.1074/jbc.272.13.8581 9079689

[33] Flamme I, Fröhlich T, Von Reutern M, et al. HRF, a putative basic helix-loop-helix-pas-domain transcription factor is closely related to hypoxia-inducible factor-1α and developmentally expressed in blood vessels. Mech Dec. 1997;63(1):51–60. doi: 10.1016/S0925-4773(97)00674-6 9178256

[34] Wang GL, Jiang BH, Rue EA, et al. Hypoxia-inducible factor 1 is a basic-helix-loop-helix-pas heterodimer regulated by cellular 02 tension (dioxin receptor/erythropoietin/hypoxia/transcription). Genetics. 1995;92:5510–5514. 10.1073/pnas.92.12.5510 PMC41725 7539918

[35] Loboda A, Jozkowicz A, Dulak J. HIF-1 and HIF-2 transcription factors - similar but not identical. Mol Cells. 2010;29(5):435–442. doi: 10.1007/s10059-010-0067-2 20396958

[36] Yang J, Zhang L, Erbel PJA, et al. Functions of the Per/ARNT/Sim domains of the hypoxia-inducible factor. J Biol Chem. 2005;280(43):36047–36054. doi: 10.1074/jbc.M501755200 16129688

[37] Erbel PJA, Card PB, Karakuzu O, et al. Structural basis for PAS domain heterodimerization in the basic helix–loop–helix-pas transcription factor hypoxia-inducible factor. Proc Natl Acad Sci U S A. 2003;100(26):15504. doi: 10.1073/pnas.2533374100 14668441 PMC307597

[38] Chapman-Smith A, Lutwyche JK, Whitelaw ML. Contribution of the Per/Arnt/Sim (PAS) domains to DNA binding by the basic helix-loop-helix PAS transcriptional regulators. J Biol Chem. 2004;279(7):5353–5362. doi: 10.1074/jbc.M310041200 14638687

[39] Semenza GL, Agani F, Booth G, et al. Structural and functional analysis of hypoxia-inducible factor 1. Kidney Int. 1997;51(2):553–555. doi: 10.1038/ki.1997.77 9027737

[40] Semenzas GL, Roth PH, Fang HM, et al. Transcriptional regulation of genes encoding glycolytic enzymes by hypoxia-inducible factor 1. J Biol Chem. 1994;269(38):23757–23763. doi: 10.1016/S0021-9258(17)31580-6 8089148

[41] Liu Y, Cox SR, Morita T, et al. Hypoxia regulates vascular endothelial growth factor gene expression in endothelial cells. Circ Res. 1995;77(3):638–643. doi: 10.1161/01.RES.77.3.638 7641334

[42] Huang LE, Gu J, Schau M, et al. Regulation of hypoxia-inducible factor 1α is mediated by an O2-dependent degradation domain via the ubiquitin-proteasome pathway. Proc Natl Acad Sci U S A. 1998;95(14):7987. doi: 10.1073/pnas.95.14.7987 9653127 PMC20916

[43] Freedman SJ, Sun ZYJ, Poy F, et al. Structural basis for recruitment of CBP/p300 by hypoxia-inducible factor-1α. Proc Natl Acad Sci U S A. 2002;99(8):5367–5372. doi: 10.1073/pnas.082117899 11959990 PMC122775

[44] Hu CJ, Sataur A, Wang L, et al. The N-Terminal transactivation domain confers target gene specificity of hypoxia-inducible factors HIF-1α and HIF-2α. Mol Biol Cell. 2007;18(11):4528. doi: 10.1091/mbc.e06-05-0419 17804822 PMC2043574

[45] Jiang BH, Zheng JZ, Leung SW, et al. Transactivation and inhibitory domains of hypoxia-inducible Factor 1α. J. Biol. Chem Journal Of Biological Chemistry. 1997;272(31):19253–19260. doi: 10.1074/jbc.272.31.19253 9235919

[46] Galbraith MD, Allen MA, Bensard CL, et al. HIF1A employs CDK8-mediator to stimulate RNAPII elongation in response to hypoxia. Cell. 2013;153(6):1327. doi: 10.1016/j.cell.2013.04.048 23746844 PMC3681429

[47] Perez-Perri JI, Dengler VL, Audetat KA, et al. The TIP60 complex is a conserved coactivator of HIF1A. Cell Rep. 2016;16(1):37. doi: 10.1016/j.celrep.2016.05.082 27320910 PMC4957981

[48] Gu YZ, Moran SM, Hogenesch JB, et al. Molecular characterization and chromosomal localization of a third α-class hypoxia inducible factor subunit, HIF3α. Gene Expr. 1998;7(3):205. 9840812 PMC6151950

[49] Depping R, Steinhoff A, Schindler SG, et al. Nuclear translocation of hypoxia-inducible factors (HIFs): involvement of the classical importin α/β pathway. Biochim et Biophys Acta (BBA) - Mol Cell Res. 2008;1783(3):394–404. doi: 10.1016/j.bbamcr.2007.12.006 18187047

[50] Bruick RK, McKnight SL. A conserved family of prolyl-4-hydroxylases that modify HIF. Sci. 2001;294(5545):1337–1340. doi: 10.1126/science.1066373 11598268

[51] Fong GH, Takeda K. Role and regulation of prolyl hydroxylase domain proteins. Cell Death Differ. 2008;15(4):635–641. doi: 10.1038/cdd.2008.10 18259202

[52] Aprelikova O, Chandramouli GVR, Wood M, et al. Regulation of HIF prolyl hydroxylases by hypoxia-inducible factors. J Cell Biochem. 2004;92(3):491–501. doi: 10.1002/jcb.20067 15156561

[53] Appelhoff RJ, Tian YM, Raval RR, et al. Differential function of the prolyl Hydroxylases PHD1, PHD2, and PHD3 in the regulation of hypoxia-inducible factor. J Biol Chem. 2004;279(37):38458–38465. doi: 10.1074/jbc.M406026200 15247232

[54] Hon WC, Wilson MI, Harlos K, et al. Structural basis for the recognition of hydroxyproline in HIF-1α by pVHL. Nature. 2002;417(6892):975–978. doi: 10.1038/nature00767 12050673

[55] Chowdhury R, McDonough MA, Mecinović J, et al. Structural basis for binding of hypoxia-inducible factor to the oxygen-sensing prolyl hydroxylases. Structure. 2009;17(7):981–989. doi: 10.1016/j.str.2009.06.002 19604478

[56] Kamura T, Sato S, Iwai K, et al. Activation of HIF1α ubiquitination by a reconstituted von hippel-lindau (VHL) tumor suppressor complex. Proc Natl Acad Sci U S A. 2000;97(19):10430–10435. doi: 10.1073/pnas.190332597 10973499 PMC27041

[57] Cardote TAF, Gadd MS, Ciulli A. Crystal structure of the Cul2-Rbx1-EloBC-vhl ubiquitin ligase complex. Structure. 2017;25(6):901. doi: 10.1016/j.str.2017.04.009 28591624 PMC5462531

[58] Haase VH. The VHL tumor suppressor: master regulator of HIF. CPD. 2009;15(33):3885–3903. doi: 10.2174/138161209789649394 PMC3622710 19671042

[59] Kamura T, Sato S, Iwai K, et al. Activation of HIF1α ubiquitination by a reconstituted von hippel-lindau (VHL) tumor suppressor complex. Proc Natl Acad Sci USA. 2000;97(19):10430–10435. doi: 10.1073/pnas.190332597 10973499 PMC27041

[60] Maxwell PH, Wlesener MS, Chang GW, et al. The tumour suppressor protein VHL targets hypoxia-inducible factors for oxygen-dependent proteolysis. Nature. 1999;399(6733):271–275. doi: 10.1038/20459 10353251

[61] Jeong JW, Bae MK, Ahn MY, et al. Regulation and destabilization of HIF-1α by ARD1-mediated acetylation. Cell. 2002;111(5):709–720. doi: 10.1016/S0092-8674(02)01085-1 12464182

[62] Elkins JM, Hewitson KS, McNeill LA, et al. Structure of factor-inhibiting hypoxia-inducible factor (HIF) reveals mechanism of oxidative modification of HIF-1α. J Biol Chem. 2002;278(3):1802–1806. 12446723 10.1074/jbc.C200644200

[63] Mahon PC, Hirota K, Semenza GL. FIH-1: a novel protein that interacts with HIF-1α and VHL to mediate repression of HIF-1 transcriptional activity. Genes Dev. 2001;15(20):2675. doi: 10.1101/gad.924501 11641274 PMC312814

[64] Lando D, Peet DJ, Gorman JJ, et al. FIH-1 is an asparaginyl hydroxylase enzyme that regulates the transcriptional activity of hypoxia-inducible factor. Genes Dev. 2002;16(12):1466. doi: 10.1101/gad.991402 12080085 PMC186346

[65] Elkins JM, Hewitson KS, McNeill LA, et al. Structure of factor-inhibiting hypoxia-inducible factor (HIF) reveals mechanism of oxidative modification of HIF-1α. Journal Of Biological Chemistry. 2002;278(3):1802–1806. doi: 10.1074/jbc.C200644200 12446723

[66] Bracken CP, Fedele AO, Linke S, et al. Cell-specific regulation of hypoxia-inducible factor (hif)-1α and HIF-2α stabilization and transactivation in a graded oxygen environment. J Biol Chem. 2006;281(32):22575–22585. doi: 10.1074/jbc.M600288200 16760477

[67] Liu YV, Baek JH, Zhang H, et al. RACK1 competes with HSP90 for binding to HIF-1α and is required for O2-independent and HSP90 inhibitor-induced degradation of HIF-1α. Mol Cell. 2007;25(2):207–217. doi: 10.1016/j.molcel.2007.01.001 17244529 PMC2563152

[68] Ravi R, Mookerjee B, Bhujwalla ZM, et al. Regulation of tumor angiogenesis by p53-induced degradation of hypoxia-inducible factor 1α. Genes Dev. 2000;14(1):34–44. doi: 10.1101/gad.14.1.34 10640274 PMC316350

[69] Mv B, Wg A, Ly R, et al. p53 inhibits hypoxia-inducible factor-stimulated transcription. J. Biol. Chem Journal Of Biological Chemistry. 1998;273(20):11995–11998. doi: 10.1074/jbc.273.20.11995 9575138

[70] Koh MY, Darnay BG, Powis G. Hypoxia-associated factor, a novel E3-ubiquitin ligase, binds and ubiquitinates hypoxia-inducible factor 1α, leading to its oxygen-independent degradation. Mol Cell Biol. 2008;28(23):7081. doi: 10.1128/MCB.00773-08 18838541 PMC2593390

[71] Bento CF, Fernandes R, Ramalho J, et al. The chaperone-dependent ubiquitin ligase CHIP targets HIF-1α for degradation in the presence of Methylglyoxal. PLoS One. 2010;5(11):e15062. doi: 10.1371/journal.pone.0015062 21124777 PMC2993942

[72] Luo W, Zhong J, Chang R, et al. Hsp70 and CHIP selectively mediate ubiquitination and degradation of hypoxia-inducible Factor (hif)-1α but not HIF-2α. J Biol Chem. 2010;285(6):3651–3663. doi: 10.1074/jbc.M109.068577 19940151 PMC2823506

[73] Duan C. Hypoxia-inducible factor 3 biology: complexities and emerging themes. Am J Physiol Cell Physiol. 2016;310(4):C260–C269. doi: 10.1152/ajpcell.00315.2015 26561641

[74] Leekl RD, R L, Sb F, et al. Association of tumour necrosis factor alpha and its receptors with thymidine phosphorylase expression in invasive breast carcinoma. Br J Cancer. 1998 12;77(12):2246–2251. doi: 10.1038/bjc.1998.373 9649140 PMC2150409

[75] Onita T, Ji P, Xuan J, et al. Hypoxia-induced, perinecrotic expression of endothelial per-ARNT-Sim domain protein-1/hypoxia-inducible factor-2alpha correlates with tumor progression, vascularization, and focal macrophage infiltration in bladder cancer. Clin Cancer Res. 2002;8(2):471–480. 11839666

[76] Wiesener MS, Jürgensen JS, Rosenberger C, et al. Widespread, hypoxia-inducible expression of HIF-2α in distinct cell populations of different organs. Faseb J. 2003;17(2):271–273. doi: 10.1096/fj.02-0445fje 12490539

[77] Iyer NV, Kotch LE, Agani F, et al. Cellular and developmental control of O2 homeostasis by hypoxia-inducible factor 1α. Genes Dev. 1998;12(2):149–162. doi: 10.1101/gad.12.2.149 9436976 PMC316445

[78] Tian H, Hammer RE, Matsumoto AM, et al. The hypoxia-responsive transcription factor EPAS1 is essential for catecholamine homeostasis and protection against heart failure during embryonic development. Genes Dev. 1998;12(21):3320–3324. doi: 10.1101/gad.12.21.3320 9808618 PMC317225

[79] Cerychova R, Pavlinkova G. HIF-1, metabolism, and diabetes in the embryonic and adult heart. Front Endocrinol (Lausanne). 2018;9(AUG):460. doi: 10.3389/fendo.2018.00460 30158902 PMC6104135

[80] Keith B, Johnson RS, Celeste Simon M. HIF1α and HIF2α: sibling rivalry in hypoxic tumour growth and progression. Nat Rev Cancer. 2012;12(1):9–22. doi: 10.1038/nrc3183 PMC3401912 22169972

[81] Hu CJ, Wang LY, Chodosh LA, et al. Differential roles of hypoxia-inducible factor 1α (HIF-1α) and HIF-2α in hypoxic gene regulation. Mol Cell Biol. 2003;23(24):9361–9374. doi: 10.1128/MCB.23.24.9361-9374.2003 14645546 PMC309606

[82] Raval RR, Lau W, Tran MGB, et al. Contrasting properties of hypoxia-inducible factor 1 (HIF-1) and HIF-2 in von hippel-lindau-associated renal cell carcinoma. Mol Cell Biol. 2005;25(13):5675–5686. doi: 10.1128/MCB.25.13.5675-5686.2005 15964822 PMC1157001

[83] Wang V, Davis DA, Haque M, et al. Differential gene up-regulation by hypoxia-inducible factor-1α and hypoxia-inducible factor-2α in HEK293T cells. Cancer Res. 2005;65(8):3299–3306. doi: 10.1158/0008-5472.CAN-04-4130 15833863

[84] Grabmaier K, De Weijert MCA, Verhaegh GW, Schalken JA, Oosterwijk E. Strict regulation of CAIX(G250/MN) by HIF-1alpha in clear cell renal cell carcinoma. Oncogene. 2004;23(33):5624–5631. doi: 10.1038/sj.onc.1207764 15184875

[85] Hu CJ, Iyer S, Sataur A, et al. Differential regulation of the transcriptional activities of hypoxia-inducible factor 1 alpha (HIF-1α) and HIF-2α in stem cells. Mol Cell Biol. 2006;26(9):3514. doi: 10.1128/MCB.26.9.3514-3526.2006 16611993 PMC1447431

[86] Downes NL, Laham-Karam N, Kaikkonen MU, et al. Differential but complementary HIF1α and HIF2α transcriptional regulation. Mol Ther. 2018;26(7):1735–1745. doi: 10.1016/j.ymthe.2018.05.004 29843956 PMC6036226

[87] Sánchez-Elsner T, Ramírez JR, Rodriguez-Sanz F, Varela E, Bernabéu C, Botella LM. A cross-talk between hypoxia and tgf-β orchestrates erythropoietin gene regulation through SP1 and smads. J Mol Biol. 2004;336(1):9–24. doi: 10.1016/j.jmb.2003.12.023 14741200

[88] Elvert G, Kappel A, Heidenreich R, et al. Cooperative interaction of hypoxia-inducible factor-2alpha (HIF-2alpha) and ets-1 in the transcriptional activation of vascular endothelial growth factor receptor-2 (flk-1). J Biol Chem. 2003;278(9):7520–7530. doi: 10.1074/jbc.M211298200 12464608

[89] Aprelikova O, Wood M, Tackett S, et al. Role of ETS transcription factors in the hypoxia-inducible factor-2 target gene selection. Cancer Res. 2006;66(11):5641–5647. doi: 10.1158/0008-5472.CAN-05-3345 16740701

[90] Keith B, Johnson RS, Simon MC. HIF1α and HIF2α: sibling rivalry in hypoxic tumor growth and progression. Nat Rev Cancer. 2012;12(1):9. doi: 10.1038/nrc3183 PMC3401912 22169972

[91] Schumacher O, Galvão DA, Taaffe DR, Chee R, Spry N, Newton RU. Exercise modulation of tumour perfusion and hypoxia to improve radiotherapy response in prostate cancer. Prostate Cancer Prostatic Dis. 2020;24(1):1–14. doi: 10.1038/s41391-020-0245-z 32632128 PMC8012204

[92] Hughes VS, Wiggins JM, Siemann DW. Tumor oxygenation and cancer therapy—then and now. Br J Radiol. 2019;92(1093):20170955. doi: 10.1259/bjr.20170955 29513032 PMC6435050

[93] Thomlinson RH, Gray LH. The histological structure of some human lung cancers and the possible implications for radiotherapy. Br J Cancer. 1955;9(4):539. doi: 10.1038/bjc.1955.55 13304213 PMC2073776

[94] Varlotto J, Stevenson MA. Anaemia, tumour hypoxemia, and the cancer patient. Int J Radiat Oncol Biol Phys. 2005;63(1):25–36. doi: 10.1016/j.ijrobp.2005.04.049 16111569

[95] Jögi A, Ehinger A, Hartman L, et al. Expression of HIF-1α is related to a poor prognosis and tamoxifen resistance in contralateral breast cancer. PLoS One. 2019;14(12):e0226150. doi: 10.1371/journal.pone.0226150 31821370 PMC6903737

[96] Kroeger N, Seligson DB, Signoretti S, et al. Poor prognosis and advanced clinicopathological features of clear cell renal cell carcinoma (ccRCC) are associated with cytoplasmic subcellular localisation of hypoxia inducible factor-2α. Eur J Cancer. 2014;50(8):1531–1540. doi: 10.1016/j.ejca.2014.01.031 24565854

[97] Chen L, Shi Y, Yuan J, et al. HIF-1 alpha overexpression correlates with poor overall survival and disease-free survival in gastric cancer patients post-gastrectomy. PLoS One. 2014;9(3):e90678. doi: 10.1371/journal.pone.0090678 24614305 PMC3948685

[98] Huang ZY, Zhang LH, Zhao C, et al. High HIF-1α expression predicts poor prognosis of patients with colon adenocarcinoma. Int J Clin Exp Pathol. 2018;11(12):5635. 31949650 PMC6963075

[99] Zhong HZ, De Marzo AM, Laughner E, et al. Overexpression of hypoxia-inducible factor 1alpha in common human cancers and their metastases. Cancer Res. 1999;59(12):5830–5835. 10582706

[100] Maher ER, Neumann HPH, Richard S. von Hippel-Lindau Disease: a clinical and scientific review. Eur J Hum Genet. 2011;19(6):617–623. doi: 10.1038/ejhg.2010.175 21386872 PMC3110036

[101] Varshney N, Kebede AA, Owusu-Dapaah H, et al. A review of Von Hippel-Lindau syndrome. J Kidney Cancer VHL. 2017;4(3):20. doi: 10.15586/jkcvhl.2017.88 28785532 PMC5541202

[102] Kim E, Zschiedrich S. Renal cell carcinoma in von Hippel–Lindau disease—from tumor genetics to novel therapeutic strategies. Front Pediatr. 2018;6:1. doi: 10.3389/fped.2018.00016 29479523 PMC5811471

[103] Ravi R, Mookerjee B, Bhujwalla ZM, et al. Regulation of tumor angiogenesis by p53-induced degradation of hypoxia-inducible factor 1α. Genes Dev. 2000;14(1):34. doi: 10.1101/gad.14.1.34 10640274 PMC316350

[104] Lee YM, Lim JH, Chun YS, et al. Nutlin-3, an Hdm2 antagonist, inhibits tumor adaptation to hypoxia by stimulating the fih-mediated inactivation of HIF-1alpha. Carcinogenesis. 2009;30(10):1768–1775. doi: 10.1093/carcin/bgp196 19696166

[105] Nieminen AL, Qanungo S, Schneider EA, Jiang BH, Agani FH. Mdm2 and HIF-1alpha interaction in tumor cells during hypoxia. J Cell Physiol. 2005;204(2):364–369. doi: 10.1002/jcp.20406 15880652

[106] LaRusch GA, Jackson MW, Dunbar JD, Warren RS, Donner DB, Mayo LD. Nutlin3 blocks vascular endothelial growth factor induction by preventing the interaction between hypoxia inducible factor 1alpha and Hdm2. Cancer Res. 2007;67(2):450–454. doi: 10.1158/0008-5472.CAN-06-2710 17234751

[107] Zundel W, Schindler C, Haas-Kogan D, et al. Loss of PTEN facilitates HIF-1-mediated gene expression. Genes Dev. 2000;14(4):391. doi: 10.1101/gad.14.4.391 10691731 PMC316386

[108] Hudson CC, Liu M, Chiang GG, et al. Regulation of hypoxia-inducible factor 1α expression and function by the mammalian target of rapamycin. Mol Cell Biol. 2002;22(20):7004. doi: 10.1128/MCB.22.20.7004-7014.2002 12242281 PMC139825

[109] Zhong H, Chiles K, Feldser D, et al. Modulation of hypoxia-inducible factor 1 alpha expression by the epidermal growth factor/phosphatidylinositol 3-kinase/PTEN/akt/frap pathway in human prostate cancer cells: implications for tumor angiogenesis and therapeutics. Cancer Res. 2000;60(6):1541–1545. 10749120

[110] Dodd KM, Yang J, Shen MH, et al. mTORC1 drives HIF-1α and VEGF-A signalling via multiple mechanisms involving 4E-BP1, S6K1 and STAT3. Oncogene. 2014;34(17):2239–2250. doi: 10.1038/onc.2014.164 24931163 PMC4172452

[111] Saraon P, Pathmanathan S, Snider J, et al. Receptor tyrosine kinases and cancer: oncogenic mechanisms and therapeutic approaches. Oncogene. 2021;40(24):4079–4093. doi: 10.1038/s41388-021-01841-2 34079087

[112] Glück AA, Aebersold DM, Zimmer Y, Medová M. Interplay between receptor tyrosine kinases and hypoxia signaling in cancer. Int J Biochem Cell Biol. 2015;62:101–114. doi: 10.1016/j.biocel.2015.02.018 25747905

[113] Iqbal N, Iqbal N. Human epidermal growth factor receptor 2 (HER2) in cancers: overexpression and therapeutic implications. Mol Biol Int. 2014;2014:1–9. doi: 10.1155/2014/852748 PMC4170925 25276427

[114] Yen L, You XL, Al Moustafa AE, et al. Heregulin selectively upregulates vascular endothelial growth factor secretion in cancer cells and stimulates angiogenesis. Oncogene. 2000;19(31):3460–3469. doi: 10.1038/sj.onc.1203685 10918604

[115] Bagheri-Yarmand R, Vadlamudi RK, Wang RA, et al. Vascular endothelial growth factor up-regulation via p21-activated kinase-1 signaling regulates heregulin-β1-mediated angiogenesis. J Biol Chem. 2000;275(50):39451–39457. doi: 10.1074/jbc.M006150200 10967114

[116] Laughner E, Taghavi P, Chiles K, et al. HER2 (neu) signaling increases the rate of hypoxia-inducible factor 1α (HIF-1α) synthesis: novel mechanism for HIF-1-Mediated vascular endothelial growth factor expression. Mol Cell Biol. 2001;21(12):3995. doi: 10.1128/MCB.21.12.3995-4004.2001 11359907 PMC87062

[117] Li YM, Zhou BP, Deng J, et al. A hypoxia-independent hypoxia-inducible factor-1 activation pathway induced by phosphatidylinositol-3 kinase/Akt in HER2 overexpressing cells. Cancer Res. 2005;65(8):3257–3263. doi: 10.1158/0008-5472.CAN-04-1284 15833858

[118] Zhang Z, Yao L, Yang J, Wang Z, Du G. PI3K/Akt and HIF-1 signaling pathway in hypoxia-ischemia. Mol Med Rep. 2018;18(4):3547. doi: 10.3892/mmr.2018.9375 30106145 PMC6131612

[119] Tian Y, Zhao L, Gui Z, et al. PI3K/AKT signaling activates HIF1α to modulate the biological effects of invasive breast cancer with microcalcification. NPJ Breast Cancer. 2023;9(1):1–12. doi: 10.1038/s41523-023-00598-z 37957150 PMC10643473

[120] Jarman EJ, Ward C, Turnbull AK, et al. HER2 regulates HIF-2α and drives an increased hypoxic response in breast cancer. Breast Cancer Res. 2019;21(1). doi: 10.1186/s13058-019-1097-0 PMC6343358 30670058

[121] Koh MY, Powis G. Passing the baton: the HIF switch. Trends Biochem Sci. 2012;37(9):364–372. doi: 10.1016/j.tibs.2012.06.004 22818162 PMC3433036

[122] Keith B, Johnson RS, Simon MC. HIF1α and HIF2α: sibling rivalry in hypoxic tumour growth and progression. Nat Rev Cancer. 2011;12(1):9–22. doi: 10.1038/nrc3183 22169972 PMC3401912

[123] Koh MY, Lemos R, Liu X, Powis G. The hypoxia-associated Factor switches cells from HIF-1α– to HIF-2α–dependent signaling promoting stem cell characteristics, aggressive tumor growth and invasion. Cancer Res. 2011;71(11):4015. doi: 10.1158/0008-5472.CAN-10-4142 21512133 PMC3268651

[124] Liu YV, Semenza GL. RACK1 vs. HSP90: competition for HIF-1α degradation vs. Stabilization. Cell Cycle. 2007;6(6):656–659. doi: 10.4161/cc.6.6.3981 17361105

[125] Chen R, Dioum EM, Hogg RT, et al. Hypoxia increases sirtuin 1 expression in a hypoxia-inducible factor-dependent manner. J Biol Chem. 2011;286(16):13869–13878. doi: 10.1074/jbc.M110.175414 21345792 PMC3077588

[126] Hagen T, Taylor CT, Lam F, Moncada S. Redistribution of intracellular oxygen in hypoxia by nitric oxide: effect on HIF1alpha. Science. 2003;302(5652):1975–1978. doi: 10.1126/science.1088805 14671307

[127] Jaśkiewicz M, Moszyńska A, Króliczewski J, et al. The transition from HIF-1 to HIF-2 during prolonged hypoxia results from reactivation of PHDs and HIF1A mRNA instability. Cell Mol Biol Lett. 2022;27(1). doi: 10.1186/s11658-022-00408-7 PMC9730601 36482296

[128] Zhang H, Pu J, Qi T, et al. MicroRNA-145 inhibits the growth, invasion, metastasis and angiogenesis of neuroblastoma cells through targeting hypoxia-inducible factor 2 alpha. Oncogene. 2012;33(3):387–397. doi: 10.1038/onc.2012.574 23222716

[129] Qu H, Zheng L, Song H, et al. microRNA-558 facilitates the expression of hypoxia-inducible factor 2 alpha through binding to 5'-untranslated region in neuroblastoma. Oncotarget. 2016;7(26):40657–40673. doi: 10.18632/oncotarget.9813 27276678 PMC5130034

[130] Serocki M, Bartoszewska S, Janaszak-Jasiecka A, et al. miRNAs regulate the HIF switch during hypoxia: a novel therapeutic target. Angiogenesis. 2018;21(2):183. doi: 10.1007/s10456-018-9600-2 29383635 PMC5878208

[131] Talks KL, Turley H, Gatter KC, et al. The expression and distribution of the hypoxia-inducible factors HIF-1α and HIF-2α in normal human tissues, cancers, and tumor-associated macrophages. Am J Pathol. 2000;157(2):411–421. doi: 10.1016/S0002-9440(10)64554-3 10934146 PMC1850121

[132] Kondo K, Kim WY, Lechpammer M, Kaelin WG, Kemp C. Inhibition of HIF2α is sufficient to suppress pVHL-defective tumor growth. PLoS Biol. 2003;1(3):e83. doi: 10.1371/journal.pbio.0000083 14691554 PMC300692

[133] Krieg M, Haas R, Brauch H, Acker T, Flamme I, Plate KH. Up-regulation of hypoxia-inducible factors HIF-1alpha and HIF-2alpha under normoxic conditions in renal carcinoma cells by von hippel-lindau tumor suppressor gene loss of function. Oncogene. 2000;19(48):5435–5443. doi: 10.1038/sj.onc.1203938 11114720

[134] Raval RR, Lau KW, Tran MGB, et al. Contrasting properties of hypoxia-inducible factor 1 (HIF-1) and HIF-2 in von hippel-lindau-associated renal cell carcinoma. Mol Cell Biol. 2005;25(13):5675. doi: 10.1128/MCB.25.13.5675-5686.2005 15964822 PMC1157001

[135] Li Y, Sun QD XX, Dai MS. Molecular crosstalk between MYC and HIF in cancer. Front Cell Dev Biol. 2020;8:590576. doi: 10.3389/fcell.2020.590576 33251216 PMC7676913

[136] Gordan JD, Bertout JA, Hu CJ, et al. HIF-2α promotes hypoxic cell proliferation by enhancing c-myc transcriptional activity. Cancer Cell. 2007;11(4):335. doi: 10.1016/j.ccr.2007.02.006 17418410 PMC3145406

[137] Gordan JD, Lal P, Dondeti VR, et al. Hif-α effects on c-myc distinguish two subtypes of sporadic VHL-Deficient clear cell renal carcinoma. Cancer Cell. 2008;14(6):435–446. doi: 10.1016/j.ccr.2008.10.016 19061835 PMC2621440

[138] Keith B, Johnson RS, Simon MC. HIF1 α and HIF2 α: sibling rivalry in hypoxic tumour growth and progression. Nat Rev Cancer. 2012;12(1):9–22. doi: 10.1038/nrc3183 PMC3401912 22169972

[139] Slawski J, Jaśkiewicz M, Barton A, et al. Regulation of the HIF switch in human endothelial and cancer cells. Eur J Cell Biol. 2024;103(2):151386. doi: 10.1016/j.ejcb.2024.151386 38262137

[140] Shen C, Beroukhim R, Schumacher SE, et al. Genetic and functional studies implicate HIF1a as a 14q kidney cancer suppressor gene. Cancer Discov. 2011;1(3):222–235. doi: 10.1158/2159-8290.CD-11-0098 22037472 PMC3202343

[141] Koshiji M, Kageyama Y, Pete EA, et al. HIF-1α induces cell cycle arrest by functionally counteracting Myc. Embo J. 2004;23(9):1949–1956. doi: 10.1038/sj.emboj.7600196 15071503 PMC404317

[142] Sato Y, Yoshizato T, Shiraishi Y, et al. Integrated molecular analysis of clear-cell renal cell carcinoma. Nat Genet. 2013;45(8):860–867. doi: 10.1038/ng.2699 23797736

[143] Schödel J, Oikonomopoulos S, Ragoussis J, et al. High-resolution genome-wide mapping of hif-binding sites by ChIP-seq. Blood. 2011;117(23):e207–e217. doi: 10.1182/blood-2010-10-314427 21447827 PMC3374576

[144] Hoefflin R, Harlander S, Schäfer S, et al. HIF-1α and HIF-2α differently regulate tumour development and inflammation of clear cell renal cell carcinoma in mice. Nat Commun. 2020;11(1):1–21. doi: 10.1038/s41467-020-17873-3 32807776 PMC7431415

[145] Schokrpur S, Hu J, Moughon DL, et al. CRISPR-Mediated VHL knockout generates an improved Model for metastatic renal cell carcinoma. Sci Rep. 2016;6(1):6. doi: 10.1038/srep29032 27358011 PMC4928183

[146] Klatte T, Seligson DB, Riggs SB, et al. Hypoxia-inducible factor 1 alpha in clear cell renal cell carcinoma. Clin Cancer Res. 2007;13(24):7388–7393. doi: 10.1158/1078-0432.CCR-07-0411 18094421

[147] Sowter HM, Ratcliffe PJ, Watson P, Greenberg AH, Harris AL. HIF-1-dependent regulation of hypoxic induction of the cell death factors BNIP3 and NIX in human tumors. Cancer Res. 2001;61(18):6669–6673. 11559532

[148] Guo K, Searfoss G, Krolikowski D, et al. Hypoxia induces the expression of the pro-apoptotic gene BNIP3. Cell Death Diff. 2001;8(4):367–376. 10.1038/sj.cdd.4400810 11550088

[149] Acker T, Diez-Juan A, Aragones J, et al. Genetic evidence for a tumor suppressor role of HIF-2alpha. Cancer Cell. 2005;8(2):131–141. doi: 10.1016/j.ccr.2005.07.003 16098466

[150] Andrysik Z, Bender H, Galbraith MD, et al. Multi-omics analysis reveals contextual tumor suppressive and oncogenic gene modules within the acute hypoxic response. Nat Commun. 2021;12(1). doi: 10.1038/s41467-021-21687-2 PMC7925689 33654095

[151] Smith MC, Gestwicki JE. Features of protein-protein interactions that translate into potent inhibitors: topology, surface area and affinity. Expert Rev Mol Med. 2012;14:e16. doi: 10.1017/erm.2012.10 22831787 PMC3591511

[152] Cukuroglu E, Engin HB, Gursoy A, Keskin O. Hot spots in protein-protein interfaces: towards drug discovery. Prog Biophys Mol Biol. 2014;116(2–3):165–173. doi: 10.1016/j.pbiomolbio.2014.06.003 24997383

[153] Greenberger LM, Horak ID, Filpula D, et al. A RNA antagonist of hypoxia-inducible factor-1α, EZN-2968, inhibits tumor cell growth. Mol Cancer Ther. 2008;7(11):3598–3608. doi: 10.1158/1535-7163.MCT-08-0510 18974394

[154] Patnaik A, Chiorean EG, Tolcher A, et al. EZN-2968, a novel hypoxia-inducible factor-1α (HIF-1α) messenger ribonucleic acid (mRNA) antagonist: results of a phase I, pharmacokinetic (PK), dose-escalation study of daily administration in patients (pts) with advanced malignancies. J Clin Oncol. 2009;27(15):2564–2564. doi: 10.1200/jco.2009.27.15_suppl.2564

[155] Jeong W, Rapisarda A, Park SR, et al. Pilot trial of EZN-2968, an antisense oligonucleotide inhibitor of hypoxia-inducible factor-1 alpha (HIF-1α), in patients with refractory solid tumors. Cancer Chemother Pharmacol. 2014;73(2):343. doi: 10.1007/s00280-013-2362-z 24292632 PMC8375568

[156] Chau NM, Rogers P, Aherne W, et al. Identification of novel small molecule inhibitors of hypoxia-inducible factor-1 that differentially block hypoxia-inducible factor-1 activity and hypoxia-inducible factor-1α induction in response to hypoxic stress and growth factors. Cancer Res. 2005;65(11):4918–4928. doi: 10.1158/0008-5472.CAN-04-4453 15930314

[157] Sapra P, Zhao H, Mehlig M, et al. Novel delivery of SN38 markedly inhibits tumor growth in xenografts, including a camptothecin-11–Refractory Model. Clin Cancer Res. 2008;14(6):1888–1896. doi: 10.1158/1078-0432.CCR-07-4456 18347192

[158] Norris RE, Shusterman S, Gore L, et al. Phase 1 evaluation of EZN-2208, a polyethylene glycol conjugate of SN38, in children adolescents and young adults with relapsed or refractory solid tumors. Pediatr Blood Cancer. 2014;61(10):1792–1797. doi: 10.1002/pbc.25105 24962521

[159] Kurzrock R, Goel S, Wheler J, et al. Safety, pharmacokinetics, and activity of EZN-2208, a novel conjugate of polyethylene glycol and SN38, in patients with advanced malignancies. Cancer. 2012;118(24):6144. doi: 10.1002/cncr.27647 22674635

[160] Osborne CRC, O’Shaughnessy J, Holmes FA, et al. Final analysis of phase II study of EZN-2208 (PEG-SN38) in metastatic breast cancer (MBC). J Clin Oncol. 2012;30(15):1017–1017. doi: 10.1200/jco.2012.30.15_suppl.101722331947

[161] Patnaik A, Papadopoulos KP, Tolcher AW, et al. Phase I dose-escalation study of EZN-2208 (PEG-SN38), a novel conjugate of poly(ethylene) glycol and SN38, administered weekly in patients with advanced cancer. Cancer Chemother Pharmacol. 2013;71(6):1499–1506. doi: 10.1007/s00280-013-2149-2 23543270 PMC3839288

[162] Garrett CR, Bekaii-Saab TS, Ryan T, et al. Randomized phase 2 study of pegylated SN-38 (EZN-2208) or irinotecan plus cetuximab in patients with advanced colorectal cancer. Cancer. 2013;119(24):4223–4230. doi: 10.1002/cncr.28358 24105075

[163] Gaur S, Chen L, Yen T, et al. Preclinical study of the cyclodextrin-polymer conjugate of camptothecin CRLX101 for the treatment of gastric cancer. Nanomed: Nanotechnol, Biol Med. 2012;8(5):721–730. doi: 10.1016/j.nano.2011.09.007 22033079

[164] Tian X, Nguyen M, Foote HP, et al. CRLX101, a nanoparticle-drug conjugate containing camptothecin, improves rectal cancer chemoradiotherapy by inhibiting DNA repair and HIF-1α. Cancer Res. 2017;77(1):112. doi: 10.1158/0008-5472.CAN-15-2951 27784746 PMC5214961

[165] Chao J, Lin J, Frankel P, et al. Pilot trial of CRLX101 in patients with advanced, chemotherapy-refractory gastroesophageal cancer. J Gastrointest Oncol. 2017;8(6):962. doi: 10.21037/jgo.2017.08.10 29299355 PMC5750185

[166] Serrano-Martínez A, Victoria-Montesinos D, García-Muñoz AM, Hernández-Sánchez P, Lucas-Abellán C, González-Louzao R. A systematic review of clinical trials on the efficacy and safety of CRLX101 Cyclodextrin-based Nanomedicine for cancer treatment. Pharmaceutics. 2023;15(7):1824. doi: 10.3390/pharmaceutics15071824 37514011 PMC10383811

[167] Weiss GJ, Chao J, Neidhart JD, et al. First-in-human phase 1/2a trial of CRLX101, a cyclodextrin-containing polymer-camptothecin nanopharmaceutical in patients with advanced solid tumor malignancies. Invest New Drugs. 2013;31(4):986–1000. doi: 10.1007/s10637-012-9921-8 23397498 PMC3774600

[168] Keefe SM, Hoffman-Censits J, Cohen RB, et al. Efficacy of the nanoparticle–drug conjugate CRLX101 in combination with bevacizumab in metastatic renal cell carcinoma: results of an investigator-initiated phase I–IIa clinical trial. Ann Oncol. 2016;27(8):1579–1585. doi: 10.1093/annonc/mdw188 27457310 PMC4959924

[169] Sanoff HK, Moon DH, Moore DT, et al. Phase I/II trial of nano-camptothecin CRLX101 with capecitabine and radiotherapy as neoadjuvant treatment for locally advanced rectal cancer. Nanomed: Nanotechnol, Biol Med. 2019;18:189–195. doi: 10.1016/j.nano.2019.02.021 PMC7881832 30858085

[170] Krasner CN, Campos SM, Young CL, et al. Sequential phase II clinical trials evaluating CRLX101 as monotherapy and in combination with bevacizumab in recurrent ovarian cancer. Gynecol Oncol. 2021;162(3):661–666. doi: 10.1016/j.ygyno.2021.07.002 34243976

[171] Terzuoli E, Puppo M, Rapisarda A, et al. Aminoflavone, a ligand of the aryl hydrocarbon receptor (AhR), inhibits HIF-1α expression in an AhR-independent fashion. Cancer Res. 2010;70(17):6837. doi: 10.1158/0008-5472.CAN-10-1075 20736373 PMC2932848

[172] Geng H, Liu Q, Xue C, et al. HIF1α protein stability is increased by acetylation at lysine 709. J Biol Chem. 2012;287(42):35496–35505. doi: 10.1074/jbc.M112.400697 22908229 PMC3471753

[173] Chen C, Wei M, Wang C, et al. The histone deacetylase HDAC1 activates HIF1α/VEGFA signal pathway in colorectal cancer. Gene. 2020;754:144851. doi: 10.1016/j.gene.2020.144851 32525044

[174] Schoepflin ZR, Shapiro IM, Risbud MV. Class I and IIa HDACs mediate HIF-1α stability through PHD2-dependent mechanism while HDAC6, a class IIb member, promotes HIF-1α transcriptional activity in nucleus pulposus cells of the intervertebral disc. J Bone Miner Res. 2016;31(6):1287. doi: 10.1002/jbmr.2787 26765925 PMC4891304

[175] Zhang C, Yang C, Feldman MJ, et al. Vorinostat suppresses hypoxia signaling by modulating nuclear translocation of hypoxia inducible factor 1 alpha. Oncotarget. 2017;8(34):56110. doi: 10.18632/oncotarget.18125 28915577 PMC5593548

[176] Qian DZ, Kachhap SK, Collis SJ, et al. Class II histone deacetylases are associated with vhl-independent regulation of hypoxia-inducible factor 1 alpha. Cancer Res. 2006;66(17):8814–8821. doi: 10.1158/0008-5472.CAN-05-4598 16951198

[177] Arnesen T, Kong X, Evjenth R, et al. Interaction between HIF-1α (ODD) and hARD1 does not induce acetylation and destabilization of HIF-1α. FEBS Lett. 2005;579(28):6428. doi: 10.1016/j.febslet.2005.10.036 16288748 PMC4505811

[178] Murray-Rust TA, Oldham NJ, Hewitson KS, Schofield CJ. Purified recombinant hARD1 does not catalyse acetylation of Lys532 of HIF-1alpha fragments in vitro. FEBS Lett. 2006;580(8):1911–1918. doi: 10.1016/j.febslet.2006.02.012 16500650

[179] Hutt DM, Roth DM, Vignaud H, et al. The histone deacetylase inhibitor, vorinostat, represses hypoxia inducible factor 1 alpha expression through translational inhibition. PLoS One. 2014;9(8):106224. doi: 10.1371/journal.pone.0106224 PMC4148404 25166596

[180] Lee YM, Kim SH, Kim HS, et al. Inhibition of hypoxia-induced angiogenesis by FK228, a specific histone deacetylase inhibitor, via suppression of HIF-1α activity. Biochem Biophys Res Commun. 2003;300(1):241–246. doi: 10.1016/S0006-291X(02)02787-0 12480550

[181] Bouyahya A, El Omari N, Bakha M, et al. Pharmacological properties of trichostatin A, focusing on the anticancer potential: a comprehensive review. Pharmaceuticals. 2022;15(10):1235. doi: 10.3390/ph15101235 36297347 PMC9612318

[182] Kang FW, Que L, Wu M, Wang ZL, Sun J. Effects of trichostatin a on HIF-1α and VEGF expression in human tongue squamous cell carcinoma cells in vitro. Oncol Rep. 2012;28(1):193–199. doi: 10.3892/or.2012.1784 22552321

[183] Katschinski DM, Le L, Schindler SG, Thomas T, Voss AK, Wenger RH. Interaction of the PAS B domain with HSP90 accelerates hypoxia-inducible factor-1α stabilization. Cell Physiol Biochem. 2004;14(4–6):351–360. doi: 10.1159/000080345 15319539

[184] Ying W, Du Z, Sun L, et al. Ganetespib, a unique triazolone-containing Hsp90 inhibitor, exhibits potent antitumor activity and a superior safety profile for cancer therapy. Mol Cancer Ther. 2012;11(2):475–484. doi: 10.1158/1535-7163.MCT-11-0755 22144665

[185] Alqawi O, Moghaddas M, Singh G. Effects of geldanamycin on HIF-1a mediated angiogenesis and invasion in prostate cancer cells. Prostate Cancer Prostatic Dis. 2006;9(2):126–135. doi: 10.1038/sj.pcan.4500852 16432534

[186] Lang SA, Klein D, Moser C, et al. Inhibition of heat shock protein 90 impairs epidermal growth factor–mediated signaling in gastric cancer cells and reduces tumor growth and vascularization in vivo. Mol Cancer Ther. 2007;6(3):1123–1132. doi: 10.1158/1535-7163.MCT-06-0628 17363505

[187] Milkiewicz M, Doyle JL, Fudalewski T, Ispanovic E, Aghasi M, Haas TL. HIF-1α and HIF-2α play a central role in stretch-induced but not shear-stress-induced angiogenesis in rat skeletal muscle. J Physiol. 2007;583(Pt 2):753. doi: 10.1113/jphysiol.2007.136325 17627993 PMC2277012

[188] Alqawi O, Moghaddas M, Singh G. Effects of geldanamycin on HIF-1α mediated angiogenesis and invasion in prostate cancer cells. Prostate Cancer Prostatic Dis. 2006;9(2):126–135. doi: 10.1038/sj.pcan.4500852 16432534

[189] Victorasso Jardim-Perassi B, Repolês Lourenço M, Mandarini Doho G, et al. Melatonin regulates angiogenic factors under hypoxia in breast cancer cell lines. Anticancer Agents Med Chem. 2016;16(3):347–358. doi: 10.2174/1871520615666150511094201 25963143

[190] Kim KJ, Choi JS, Kang I, et al. Melatonin suppresses tumor progression by reducing angiogenesis stimulated by HIF-1 in a mouse tumor model. J Pineal Res. 2013;54(3):264–270. doi: 10.1111/j.1600-079X.2012.01030.x 22924616

[191] Hwang SJ, Jung Y, Song YS, et al. Enhanced anti-angiogenic activity of novel melatonin-like agents. J Pineal Res. 2021;71(1):e12739. doi: 10.1111/jpi.12739 33955074 PMC8365647

[192] Lee K, Lee JH, Boovanahalli SK, et al. (Aryloxyacetylamino)benzoic acid analogues: a new class of hypoxia-inducible factor-1 inhibitors. J Med Chem. 2007;50(7):1675–1684. doi: 10.1021/jm0610292 17328532

[193] Lee K, Kang JE, Park SK, et al. LW6, a novel HIF-1 inhibitor, promotes proteasomal degradation of HIF-1α via upregulation of VHL in a colon cancer cell line. Biochem Pharmacol. 2010;80(7):982–989. doi: 10.1016/j.bcp.2010.06.018 20599784

[194] Lee JY, Lee K, Lee K, et al. Pharmacokinetic characterization of LW6, a novel hypoxia-inducible factor-1α (HIF-1α) inhibitor in mice. Molecules. 2021;26(8):2226. doi: 10.3390/molecules26082226 33921487 PMC8070284

[195] Kung AL, Wang S, Klco JM, Kaelin WG, Livingston DM. Suppression of tumor growth through disruption of hypoxia-inducible transcription. Nat Med. 2000;6(12):1335–1340. doi: 10.1038/82146 11100117

[196] Dames SA, Martinez-Yamout M, De Guzman RN, et al. Structural basis for hif-1α/CBP recognition in the cellular hypoxic response. Proc Natl Acad Sci U S A. 2002;99(8):5271–5276. doi: 10.1073/pnas.082121399 11959977 PMC122759

[197] Henchey LK, Kushal S, Dubey R, Chapman RN, Olenyuk BZ, Arora PS. Inhibition of hypoxia inducible factor 1-transcription coactivator interaction by a hydrogen bond surrogate α-helix. J Am Chem Soc. 2010;132(3):941–943. doi: 10.1021/ja9082864 20041650 PMC2810346

[198] Kushal S, Lao BB, Henchey LK, et al. Protein domain mimetics as in vivo modulators of hypoxia-inducible factor signaling. Proc Natl Acad Sci U S A. 2013;110(39):15602–15607. doi: 10.1073/pnas.1312473110 24019500 PMC3785738

[199] Lao BB, Grishagin I, Mesallati H, et al. In vivo modulation of hypoxia-inducible signaling by topographical helix mimetics. Proc Natl Acad Sci U S A. 2014;111(21):7531–7536. doi: 10.1073/pnas.1402393111 24821806 PMC4040591

[200] Jiang W, Abdulkadir S, Zhao X, et al. Inhibition of hypoxia-inducible transcription factor (HIF-1α) signaling with sulfonyl-γ-AApeptide helices. J Am Chem Soc. 2023;145(36):20009–20020. doi: 10.1021/jacs.3c06694 37665648 PMC10637359

[201] Burslem GM, Kyle HF, Breeze AL, et al. Small-molecule proteomimetic inhibitors of the HIF-1α–p300 protein–protein interaction. Chembiochem. 2014;15(8):1083–1087. doi: 10.1002/cbic.201400009 24782431 PMC4159589

[202] Cook KM, Hilton ST, Mecinovic J, et al. Epidithiodiketopiperazines block the interaction between hypoxia-inducible factor-1α (HIF-1α) and p300 by a zinc ejection mechanism. J Biol Chem. 2009;284(39):26831–26838. doi: 10.1074/jbc.M109.009498 19589782 PMC2785371

[203] Block KM, Wang H, Szabó LZ, et al. Direct inhibition of hypoxia-inducible transcription factor complex with designed dimeric epidithiodiketopiperazine. J Am Chem Soc. 2009;131(50):18078–18088. doi: 10.1021/ja807601b 20000859 PMC2796602

[204] Jayatunga MKP, Thompson S, McKee TC, et al. Inhibition of the HIF1α-p300 interaction by quinone- and indandione-mediated ejection of structural Zn(II). Eur J Med Chem. 2015;94:509–516. doi: 10.1016/j.ejmech.2014.06.006 25023609 PMC4277744

[205] Brignole C, Marimpietri D, Pastorino F, et al. Effect of Bortezomib on human neuroblastoma cell growth, Apoptosis, and angiogenesis. JNCI: J Natl Cancer Inst. 2006;98(16):1142–1157. doi: 10.1093/jnci/djj309 16912267

[206] Roccaro AM, Hideshima T, Raje N, et al. Bortezomib mediates antiangiogenesis in multiple myeloma via direct and indirect effects on endothelial cells. Cancer Res. 2006;66(1):184–191. doi: 10.1158/0008-5472.CAN-05-1195 16397231

[207] Velcade RL. Receives 2 new FDA indications: for retreatment of patients with multiple myeloma and for first-line treatment of patients with Mantle-Cell Lymphoma. Am Health Drug Benefits. 2015;8(Spec Feature):135. 26629279 PMC4665054

[208] Richardson PG, Mitsiades C, Hideshima T, Anderson KC. Bortezomib: proteasome inhibition as an effective anticancer therapy. Annu Rev Med. 2006;57(1):33–47. doi: 10.1146/annurev.med.57.042905.122625 16409135

[209] Shin DH, Chun YS, Lee DS, et al. Bortezomib inhibits tumor adaptation to hypoxia by stimulating the fih-mediated repression of hypoxia-inducible factor-1. Blood. 2008;111(6):3131–3136. doi: 10.1182/blood-2007-11-120576 18174379

[210] Abd-Aziz N, Stanbridge EJ, Shafee N. Bortezomib attenuates HIF-1- but not HIF-2-mediated transcriptional activation. Oncol Lett. 2015;10(4):2192. doi: 10.3892/ol.2015.3545 26622817 PMC4579903

[211] Kong D, Park EJ, Stephen AG, et al. Echinomycin, a small-molecule inhibitor of hypoxia-inducible factor-1 DNA-Binding activity. Cancer Res. 2005;65(19):9047–9055. doi: 10.1158/0008-5472.CAN-05-1235 16204079

[212] Tanaka T, Yamaguchis J, Shojis K, et al. Anthracycline inhibits recruitment of hypoxia-inducible transcription factors and suppresses tumor cell migration and cardiac angiogenic response in the Host. J Biol Chem. 2012;287(42):34866. doi: 10.1074/jbc.M112.374587 22908232 PMC3471702

[213] Pang Y, Yang C, Schovanek J, et al. Anthracyclines suppress pheochromocytoma cell characteristics, including metastasis, through inhibition of the hypoxia signaling pathway. Oncotarget. 2017;8(14):22313. doi: 10.18632/oncotarget.16224 28423608 PMC5410225

[214] Henry JT, Crosson S. Ligand binding PAS domains in a genomic, cellular, and structural context. Annu Rev Microbiol. 2011;65(1):261. doi: 10.1146/annurev-micro-121809-151631 21663441 PMC3298442

[215] Scheuermann TH, Tomchick DR, Machius M, et al. Artificial ligand binding within the HIF2α PAS-B domain of the HIF2 transcription factor. Proc Natl Acad Sci U S A. 2009;106(2):450–455. doi: 10.1073/pnas.0808092106 19129502 PMC2626723

[216] Scheuermann TH, Li Q, Ma HW, et al. Allosteric inhibition of hypoxia inducible factor-2 with small molecules. Nat Chem Biol. 2013;9(4):271. doi: 10.1038/nchembio.1185 23434853 PMC3604136

[217] Rogers JL, Bayeh L, Scheuermann TH, et al. Development of inhibitors of the PAS-B domain of the HIF-2α transcription factor. J Med Chem. 2013;56(4):1739. doi: 10.1021/jm301847z 23363003 PMC3676484

[218] Nepali K, Lee HY, Liou JP. Nitro-group-containing drugs. J Med Chem. 2019;62(6):2851–2893. doi: 10.1021/acs.jmedchem.8b00147 30295477

[219] Scheuermann TH, Stroud D, Sleet CE, et al. Isoform-selective and stereoselective inhibition of hypoxia inducible factor-2. J Med Chem. 2015;58(15):5930–5941. doi: 10.1021/acs.jmedchem.5b00529 26226049

[220] Wehn PM, Rizzi JP, Dixon DD, et al. Design and activity of specific hypoxia-inducible factor-2α (HIF-2α) inhibitors for the treatment of clear cell renal cell carcinoma: discovery of clinical Candidate (s)-3-((2,2-difluoro-1-hydroxy-7-(methylsulfonyl)-2,3-dihydro-1 h-inden-4-yl)oxy)-5-fluorobenzonitrile (PT2385). J Med Chem. 2018;61(21):9691–9721. 30289716 10.1021/acs.jmedchem.8b01196

[221] Courtney KD, Infante JR, Lam ET, et al. Phase I dose-escalation trial of PT2385, a first-in-Class hypoxia-inducible Factor-2α antagonist in patients with Previously treated advanced clear cell renal cell carcinoma. J Clinc Oncol. 2018;36(9):867. doi: 10.1200/JCO.2017.74.2627 PMC5946714 29257710

[222] Xu R, Wang K, Rizzi JP, et al. 3-[(1 S,2 S,3 R)-2,3-Difluoro-1-hydroxy-7-methylsulfonylindan-4-yl]oxy-5-fluorobenzonitrile (PT2977), a Hypoxia-Inducible Factor 2α (HIF-2α) Inhibitor for the Treatment of Clear Cell Renal Cell Carcinoma. J Med Chem. 2019;62(15):6876–6893. doi: 10.1021/acs.jmedchem.9b00719 31282155

[223] Papadopoulos KP, Jonasch E, Zojwalla NJ, Wang K, Bauer TM. A first-in-human phase 1 dose-escalation trial of the oral HIF-2a inhibitor PT2977 in patients with advanced solid tumors. J Clinc Oncol. 2018;36(15_suppl):2508–2508. doi: 10.1200/JCO.2018.36.15_suppl.2508

[224] Jonasch E, Plimack ER, Bauer T, et al. A first-in-human phase I/II trial of the oral HIF-2a inhibitor PT2977 in patients with advanced RCC. Ann Oncol. 2019;30:v361–v362. doi: 10.1093/annonc/mdz249.010

[225] Jonasch E, Park EK, Thamake S, Hirmand M, Linehan WM, Srinivasan R. An open-label phase II study to evaluate PT2977 for the treatment of von Hippel-Lindau disease-associated renal cell carcinoma. J Clinc Oncol. 2019;37(7):TPS680–TPS680. doi: 10.1200/JCO.2019.37.7_suppl.TPS680

[226] Fallah J, Brave MH, Weinstock C, et al. FDA Approval Summary: Belzutifan for von Hippel-Lindau disease associated tumors. Clin Cancer Res. 2022;28(22):4843. doi: 10.1158/1078-0432.CCR-22-1054 35727604 PMC9669093

[227] Lawson K, Kelsey E, Gauthier, et al. Discovery and characterisation of AB521, a novel, potent, and selective hypoxia-inducible (hif)-2a inhibitor. Cancer Res. 2021;81(13_Supplement):1206.

[228] Eun JP, Kong D, Fisher R, Cardellina J, Shoemaker RH, Melillo G. Targeting the PAS-A domain of HIF-1 alpha for development of small molecule inhibitors of HIF-1. Cell Cycle. 2006;5(16):1847–1853. doi: 10.4161/cc.5.16.3019 16861921

[229] Lee KA, Zhang H, Qian DZ, et al. RETRACTED: acriflavine inhibits HIF-1 dimerization, tumor growth, and vascularization. Proc Natl Acad Sci U S A. 2009;106(42):17910–17915. doi: 10.1073/pnas.0909353106 19805192 PMC2764905

[230] Wu D, Potluri N, Lu J, Kim Y, Rastinejad F. Structural integration in hypoxia-inducible factors. Nature. 2015;524(7565):303–308. doi: 10.1038/nature14883 26245371

[231] Piorecka K, Kurjata J, Stanczyk WA. Acriflavine, an acridine derivative for biomedical application: current state of the art. J Med Chem. 2022;65(17):11415–11432. doi: 10.1021/acs.jmedchem.2c00573 36018000 PMC9469206

[232] Hassan S, Laryea D, Mahteme H, et al. Novel activity of acriflavine against colorectal cancer tumor cells. Cancer Sci. 2011;102(12):2206–2213. doi: 10.1111/j.1349-7006.2011.02097.x 21910782

[233] Mangraviti A, Raghavan T, Volpin F, et al. HIF-1α- Targeting Acriflavine Provides Long Term Survival and Radiological Tumor Response in Brain Cancer Therapy. Sci Rep. 2017;7(1):1–13. doi: 10.1038/s41598-017-14990-w 29097800 PMC5668269

[234] Seredinski S, Boos F, Günther S, et al. DNA topoisomerase inhibition with the HIF inhibitor acriflavine promotes transcription of lncRNAs in endothelial cells. Mol Ther Nucleic Acids. 2022;27:1023–1035. doi: 10.1016/j.omtn.2022.01.016 35228897 PMC8844413

[235] Wong CCL, Zhang H, Gilkes DM, et al. Inhibitors of Hypoxia-Inducible Factor 1 Block Breast Cancer Metastastic Niche Formation and Lung Metastasis. J Mol Med (Berl). 2012;90(7):803. doi: 10.1007/s00109-011-0855-y 22231744 PMC3437551

[236] Bulle A, Dekervel J, Libbrecht L, et al. Gemcitabine induces epithelial-to-mesenchymal transition in patient-derived pancreatic ductal adenocarcinoma xenografts. Am J Transl Res. 2019;11(2):765. 30899378 PMC6413274

[237] Cheloni G, Tanturli M, Tusa I, et al. Targeting chronic myeloid leukemia stem cells with the hypoxia-inducible factor inhibitor acriflavine. Blood. 2017;130(5):655. doi: 10.1182/blood-2016-10-745588 28576876 PMC5942867

[238] Broekgaarden M, Weijer R, Krekorian M, et al. Inhibition of hypoxia-inducible factor 1 with acriflavine sensitizes hypoxic tumor cells to photodynamic therapy with zinc phthalocyanine-encapsulating cationic liposomes. Nano Res. 2016;9(6):1639–1662. doi: 10.1007/s12274-016-1059-0

[239] Hypoxia PM. Inflammation and redox status as determinants of malignant progression of cancer stem cells. Ann Oncol. 2017;28(5):114. https://www.annalsofoncology.org/article/S0923-7534(20)37762-0/fulltext

[240] Fan J, Yang X, Bi Z. Acriflavine suppresses the growth of human osteosarcoma cells through apoptosis and autophagy. Tumour Biol. 2014;35(10):9571–9576. doi: 10.1007/s13277-014-2156-x 24961347

[241] Martí-Díaz R, Montenegro MF, Cabezas-Herrera J, Goding CR, Rodríguez-López JN, Sánchez-Del-Campo L. Acriflavine, a Potent Inhibitor of HIF-1α, Disturbs Glucose Metabolism and Suppresses ATF4-Protective Pathways in Melanoma under Non-Hypoxic Conditions. Cancers (Basel). 2020;13(1):102. doi: 10.3390/cancers13010102 33396270 PMC7795823

[242] Yin T, He S, Shen G, Wang Y. HIF-1 Dimerization Inhibitor Acriflavine Enhances Antitumor Activity of Sunitinib in Breast Cancer Model. Oncol Res. 2014;22(3):139–145. doi: 10.3727/096504014X13983417587366 26168132 PMC7838425

[243] Piorecka K, Kurjata J, Gostynski B, et al. Is acriflavine an efficient co-drug in chemotherapy?. RSC Adv. 2023;13(31):21421–21431. doi: 10.1039/D3RA02608F 37465576 PMC10350790

[244] Zargar P, Ghani E, Jalali Mashayekhi F, Ramezani A, Eftekhar E. Acriflavine enhances the antitumor activity of the chemotherapeutic drug 5-fluorouracil in colorectal cancer cells. Oncol Lett. 2018;15(6):10084. doi: 10.3892/ol.2018.8569 29928378 PMC6004650

[245] Tubbs RK, Ditmars WE, van Winkle Q. Heterogeneity of the interaction of DNA with acriflavine. J Mol Biol. 1964;9(2):545–557. doi: 10.1016/S0022-2836(64)80226-6 14202285

[246] Cardoso R, Love R, Nilsson CL, et al. Identification of Cys255 in HIF-1α as a novel site for development of covalent inhibitors of HIF-1α/ARNT PasB domain protein-protein interaction. Protein Sci. 2012;21(12):1885–1896. doi: 10.1002/pro.2172 23033253 PMC3575918

[247] Miranda E, Nordgren IK, Male AL, et al. A cyclic peptide inhibitor of HIF-1 heterodimerization that inhibits hypoxia signaling in cancer cells. J Am Chem Soc. 2013;135(28):10418. doi: 10.1021/ja402993u 23796364 PMC3715890

[248] Mistry IN, Tavassoli A. Reprogramming the transcriptional response to hypoxia with a chromosomally encoded cyclic peptide HIF-1 inhibitor. ACS Synth Biol. 2017;6(3):518–527. doi: 10.1021/acssynbio.6b00219 27978620 PMC6014682

[249] Ball AT, Mohammed S, Doigneaux C, et al. Identification and Development of Cyclic Peptide Inhibitors of Hypoxia Inducible Factors 1 and 2 That Disrupt Hypoxia-Response Signaling in Cancer Cells. J Am Chem Soc. 2023;146(13):8877–8886. doi: 10.1021/jacs.3c10508 PMC10996005 38503564

